# Neuropeptide FF (NPFF)-positive nerve cells of the human cerebral cortex and white matter in controls, selected neurodegenerative diseases, and schizophrenia

**DOI:** 10.1186/s40478-024-01792-1

**Published:** 2024-06-28

**Authors:** Diana Wiesner, Simone Feldengut, Sarah Woelfle, Tobias M. Boeckers, Albert C. Ludolph, Francesco Roselli, Kelly Del Tredici

**Affiliations:** 1 Department of Neurology, Center for Biomedical Research, Ulm University, 89081 Ulm, Germany; 2 DZNE, Ulm Site, 89081 Ulm, Germany; 3 Clinical Neuroanatomy Section, Department of Neurology, Center for Biomedical Research, Ulm University, 89081 Ulm, Germany; 4 Institute for Anatomy and Cell Biology, Ulm University, 89081 Ulm, Germany

**Keywords:** Alzheimer’s disease, Amyotrophic lateral sclerosis, Cerebral cortex, Human brain, Interneurons, Neurodegeneration, Neuropeptide FF (NPFF), NOS (type I), Pick’s disease, Schizophrenia, Somatostatin, U-fibers, White matter interstitial cells

## Abstract

We quantified and determined for the first time the distribution pattern of the neuropeptide NPFF in the human cerebral cortex and subjacent white matter. To do so, we studied *n* = 9 cases without neurological disorders and *n* = 22 cases with neurodegenerative diseases, including sporadic amyotrophic lateral sclerosis (ALS, *n* = 8), Alzheimer’s disease (AD, *n* = 8), Pick’s disease (PiD, *n* = 3), and schizophrenia (*n* = 3). NPFF-immunopositive cells were located chiefly, but not exclusively, in the superficial white matter and constituted there a subpopulation of white matter interstitial cells (WMIC): Pyramidal-like and multipolar somata predominated in the gyral crowns, whereas bipolar and ovoid somata predominated in the cortex surrounding the sulci. Their sparsely ramified axons were unmyelinated and exhibited NPFF-positive bead-like varicosities. We found significantly fewer NPFF-immunopositive cells in the gray matter of the frontal, cingulate, and superior temporal gyri of both sporadic ALS and late-stage AD patients than in controls, and significantly fewer NPFF-positive cells in the subjacent as well as deep white matter of the frontal gyrus of these patients compared to controls. Notably, the number of NPFF-positive cells was also significantly lower in the hippocampal formation in AD compared to controls. In PiD, NPFF-positive cells were present in significantly lower numbers in the gray and white matter of the cingulate and frontal gyrii in comparison to controls. In schizophrenic patients, lower wNPFF cell counts in the neocortex were significant and global (cingulate, frontal, superior temporal gyrus, medial, and inferior gyri). The precise functions of NPFF-positive cells and their relationship to the superficial corticocortical white matter U-fibers are currently unknown. Here, NPFF immunohistochemistry and expression characterize a previously unrecognized population of cells in the human brain, thereby providing a new entry-point for investigating their physiological and pathophysiological roles.

**Supplementary Information:**

The online version contains supplementary material available at 10.1186/s40478-024-01792-1.

## Introduction

The white matter interstitial cells (WMIC) of the human brain, first described by Theodor Meynert [34, 55] and representing approximately 3% of all cortical neurons (between 600 and 1100 million nerve cells) [41, 72, 73], mature and undergo axonal myelination postnatally [84]. They are most prominent in the human and primate brain, and are also present in the brains of non-primate mammals and rodents [14, 35, 54, 56, 61, 75–78, 86]. Little is known about the functional significance of WMIC or their connectivities, i.e., the sources of their afferents and the targets of their efferent connections. In humans, the physiological functions attributed to WMIC include regulation of the cerebral circulation [27, 60], sleep regulation [36], and control of the information flow through the cerebral cortex [15, 43]. However, reports also indicate that superficial WMIC and their late-myelinating axons may be implicated in the pathophysiology and pathogenesis of neurological, neurodevelopmental, and neuropsychiatric disorders, such as Alzheimer’s disease, Huntington’s disease, schizophrenia, bipolar disease, autism spectrum disorder, and epilepsy [1–4, 16, 17, 22, 23, 32, 37, 43, 45, 47, 56, 65, 66, 70, 85, 89]. Up to now, both their neurochemical heterogeneity [94] and the lack of a reliable marker with high staining efficiency in human autopsy tissue have made it difficult to obtain a more comprehensive understanding of WMIC morphology, distribution, and involvement across the spectrum of neurodegenerative diseases.

Here, we report the discovery that Neuropeptide FF (NPFF), an octapeptide belonging to an extended family of RF-amide peptides, reliably identifies a subpopulation of cells in both the white matter and gray matter of the human neo- and allocortex. NPFF was first isolated in 1985 in the bovine central nervous system (CNS) and, subsquently, in the rat CNS [39, 40, 88]. In humans, NPFF is encoded by the *NPFF* gene [63]; together with its receptors, NPFFR1 and NPFFR2, the peptide is implicated in a broad spectrum of physiological functions in the vertebrate brain, spinal cord, and periphery [18, 28, 29, 44, 48–50, 53, 68, 71, 80, 92]. These include antinoception, pruritic sensation, and regulation of endocrine and cardiovascular functions [26, 28, 29, 48–50, 53, 62].

The present report documents the number, morphological features, and distribution pattern of NPFF-positive cells in the cerebral cortex of *n* = 9 control subjects and in *n* = 22 individuals with selected neurodegenerative diseases using immunoreactions with a polyclonal antibody directed against NPFF. Here, we use the term “NPFF-positive cells” to refer to both cortical gray matter (gNPFF) and subjacent cortical white matter (wNPFF) cells as a group.

## Material and methods

### Human postmortem tissue

This study was performed in compliance with university ethics committee guidelines, the declaration of Helsinki, as well as German federal and state law governing human tissue usage. Informed written consent for autopsy was obtained from the patients or their next of kin. Postmortem tissue samples from the University of Ulm Tissue Bank were included from *n* = 9 individuals (5 females, 4 males; age 56.4 ± 24.1 years) without neurological disorders as controls, *n* = 8 patients with sporadic ALS (2 females, 6 males; age 61.1 ± 15.68 years), n = 8 patients with advanced sporadic AD (5 females, 3 males; age 80.2 ± 9.1 years), *n* = 3 patients with PiD (2 females, 1 male; 72.0 ± 1.63 years), and n = 3 patients with schizophrenia (1 female, 2 males; 63.3 ± 7.4 years). Cases were selected by gender and age such that there was one case for each gender and age group. In this manner, we were able to detect or exclude both age- and gender-specific effects [57]. Demographic and neuropathological data for all of the individuals studied are shown in Supplementary Table 1.

### Tissue fixation, embedding, and sectioning

Brains were fixed for 14–21 days by immersion in a 4% aqueous solution of formaldehyde. Afterwards, we used a macrotome to cut 1 cm thick coronal slices perpendicular to Forel’s axis from at least one hemisphere of each case. We used the hemisphere block that was cut at mid-uncal level and included anterior portions of the hippocampal formation. We also excised from two controls (Suppl. Table 1, cases 8 and 9) an additional tissue block halfway between the occipital pole and the junction of the parieto-occipital sulcus with the calcarine fissure. The sections were oriented perpendicular to the calcarine fissure and contained the striate (primary sensory area 17), parastriate (secondary sensory area 18), and peristriate (area 19) regions (Suppl. Figure 1). The tissue blocks were embedded in polyethylene glycol (PEG 1000, Merck, Carl Roth Ltd, Karlsruhe, Germany), and sectioning was performed with a tetrander (Jung, Heidelberg, Germany) at a thickness of 100 µm, as described previously [8].

### Histological staining and immunohistochemistry

Following pretreatment with performic acid, pigment-Nissl staining (aldehyde fuchsine, Morphisto, Frankfurt am Main, Germany, combined with a basophilic Nissl stain Darrow red, 211,885, Sigma-Aldrich, Steinheim, Germany) was performed on free-floating hemisphere sections for topographical orientation and to show the presence and extent of lipofuscin deposits as well as neuronal loss [5, 9]. For immunohistochemistry, hemisphere sections were treated with 10% methanol plus 10% concentrated (30%) H_2_O_2_ and 80% Tris for 30 min. Following microwave pretreatment for 30 min (NPFF, pTDP-43, SMI-31, MBP, somatostatin, calretinen) or 1 h (TDP), or pretreatment with 100% formic acid for 3 min (4G8, syn-1) to facilitate the immunoreactions, blocking with bovine serum albumin was performed to inhibit endogenous peroxidase and to prevent nonspecific binding. Next, free-floating sections were incubated for 18 h* at 20 °C using primary antibodies (Suppl. Table 2).

Additional 100 µm hemisphere sections from each case were immunostained with the following primary antibodies: (**1**) a monoclonal rabbit recombinant somatostatin antibody (7Q4T0; 1:1000, Invitrogen [Thermo Scientific], Waltham, MA, USA), (**2**) a polyclonal rabbit calretinin antibody (1:1000; Invitrogen [Thermo Scientific], Waltham, MA, USA), or (**3**) a monoclonal parvalbumin antibody (1:1000, Sigma-Aldrich/Merck, Darmstadt, Germany). From the frontal gyrus of two cases (Suppl. Table 1, cases 8 and 9), 100 µm sections were immunostained with one of the following primary antibodies: (**1**) a polyclonal calbindin antibody (1:2000, Invitrogen [Thermo Scientific] PA5-143,561, Waltham, MA, USA) or (**2**) a monoclonal nitric oxide synthase neuronal NOS-125 antibody (1:100, GeneTex GTX01921, Irvine, CA, USA). In double-immunoreactions for NPFF/TDP-43, NPFF/AT8, NPFF/syn-1, NPFF/pTDP-43, NPFF/SMI-31, NPFF/MPB, NPFF/somatostatin, NPFF/calretinin, and NPFF/parvalbumin, NPFF/calbindin, and NPFF/nNOS, the NPFF immunoreaction was visualized using the brown chromogen (DAB, D5637 Sigma, Taufkirchen, Germany) and the second immunoreaction was visualized with the blue chromogen SK-4700 (SG Substrate Kit, Vector, Newark, NJ, USA).

Subsequent to processing with a corresponding secondary biotinylated antibody (anti-mouse IgG, 1:200; Linaris) for 1.5 h, all immunoreactions were visualized with the avidin–biotin complex (ABC, Vectastain, Vector Laboratories, Burlingame, CA, USA) for 2 h and DAB. Omission of the primary antiserum resulted in non-staining. Positive as well as negative control sections were included to confirm immunostaining specificity. NPFF immunoreactions underwent counterstaining for lipofuscin pigment with aldehyde fuchsin (Morphisto, Frankfurt am Main, Germany) [5].

Tissue sections were cleared, mounted, and cover-slipped (Histomount, National Diagnostics, Atlanta, GA, USA). Histological slides were viewed and neuropathological staging performed with an Olympus BX61 microscope (Olympus Optical, Tokyo, Japan) (K.D.T.). Digital micrographs were taken with an Olympus XC50 camera using the Cell D® Imaging Software (Olympus, Münster, Germany) (K.D.T., D.W.). The program’s Extended Focal Imaging (EFI) function was used to fuse stacks of four differently focused single images into a single sharply focused image.

### Immunofluorescence (IF) and confocal imaging

Heat-induced epitope retrieval was performed by boiling the tissue sections in 0.1 M citric acid (pH 6.0) in a water bath for 30 min. After cooling to room temperature, the sections were incubated in blocking solution (3% (wt/vol) BSA plus 0.1% (vol/vol) Triton X-100 in 1 × DPBS^−/−^) under gentle agitation at room temperature for 2 h. Primary antibodies (NPFF, VGAT [vesicular GABA transporter], and gephyrin, see Suppl. Table 2) were diluted in blocking solution and incubated with the tissue under gentle agitation at 4 °C for 3 days. After rinsing 3 times with 1 × DPBS^−/−^ for 30 min, Alexa Fluor-coupled secondary antibodies were used for 2 h. Finally, all tissue sections were mounted in SlowFade Gold antifade (Thermo Fischer Scientific, Eugene, OR, USA), and z-stacks were acquired on a SPE confocal microscope (Leica Microsystems, Wetzler, Germany) with the 63 × oil objective (ACS APO, NA 1.3, WD 160 µm).

### NPFF cell quantification

**Fig. 1:**
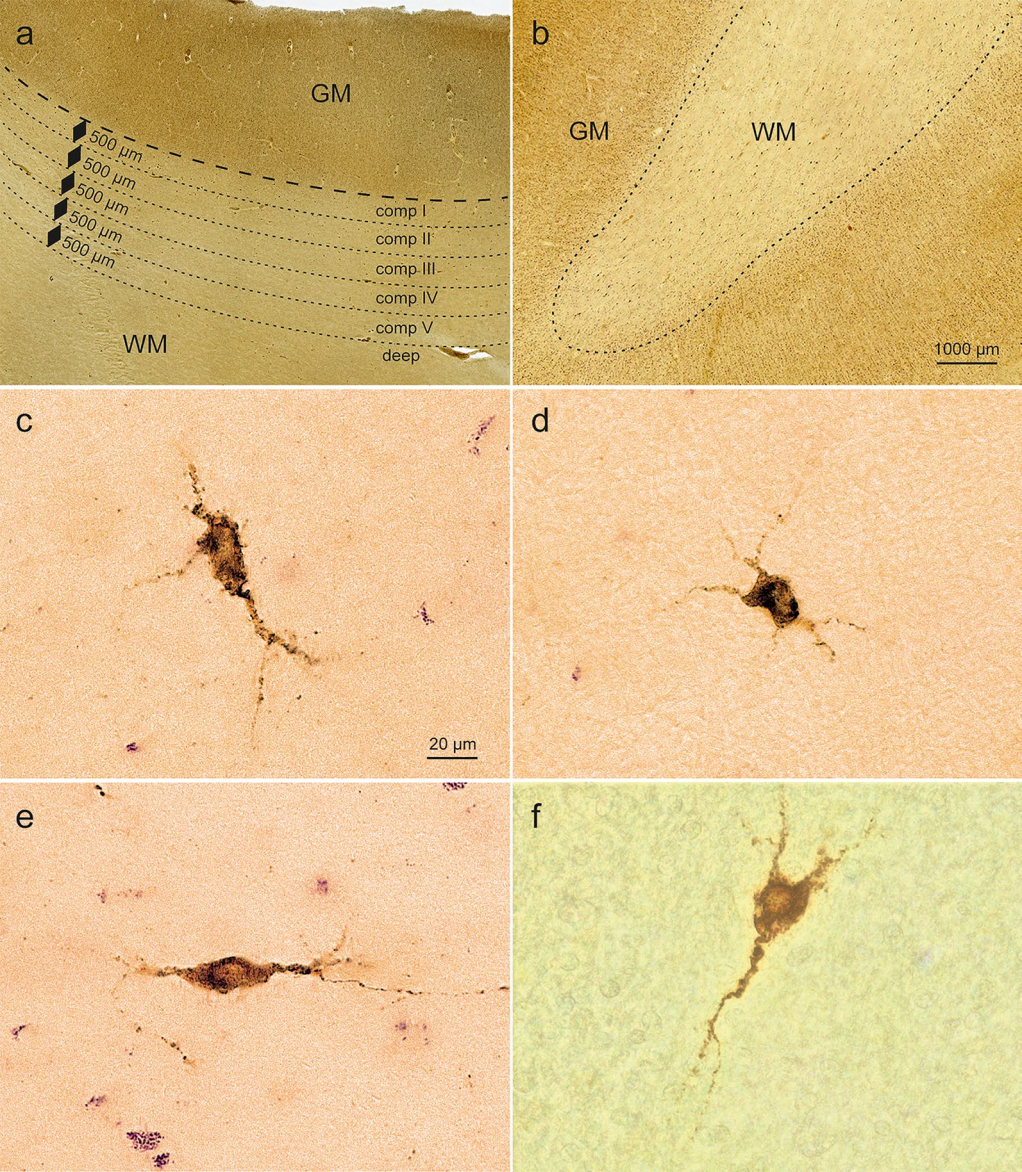
Superificial white matter compartments and white matter NPFF-immunopositive cells in a control case. **a** Detail showing gray matter (GM) und white matter (WM), including the superficial white matter compartments I bis V (*broken lines*) and deep white matter. Each white matter compartment is 500 µm in thickness. Scale bar in **b** is also valid for **a. b** Gyral crown of frontal gyrus. The density of the NPFF cells in the white matter increases with increasing proximity to the gyral crown. **c–f** Polymorph appearances of white matter NPFF-positive cells from the frontal gyrus; pyramidal-like (triangular-shaped) in **c** and** f** (see also Fig. 2f), multipolar **d**, and bipolar/fusiform **e** Scale bar in **c** also applies to **d–f**. **a–f** Case 8: male, 83 years of age (see also Suppl. Figure 1). Pigment (aldehyde fuchsin) staining plus NPFF-immunohistochemistry (brown chromogen 3,3’-diaminobenzidine tetrahydrochloride, DAB) in 100 µm polyethylene glycol-embedded (PEG) sections

The regions of interest were identified by light microscopy in immunohistochemically stained sections, according to anatomical landmarks [52] (Suppl. Figure 1). The superficial white matter region is considered to be a transition zone between the infragranular layer VI and the deep white matter [22, 24]. To distinguish the border between gray matter and superficial white matter, we performed pigment-Nissl staining on additional sections. The border was determined using the rapid decrease in neuronal density and dendritic orientation [76]. To quantify the NPFF cells in each coronal section, we further subdivided the superficial white matter region into compartments I-V at a depth of 500 µm based on Conner et al. [16] for each gyrus selected for analysis (Fig. 1a). In samples where it was not possible to classify all five WM compartments, the rating NE (not evaluated) was entered. The gyri included the cingulate gyrus, frontal gyrus, superior temporal gyrus, medial and inferior temporal gyri, and temporal allocortex (including the entorhinal region and hippocampal formation; Suppl. Figure 2a, b). Cell counts were carried out on 100 µm hemisphere sections (D.W.). Brightfield images with 4X tiles and Z-stacks of 6 µm thick optical sections were acquired with a Keyence BZ-X800 series microscope and run by the BZ-X800 analyzer software (Keyence Canada Inc., Mississauga, Ontario, Canada). For image analysis, maximal intensity images were made from 22 to 25 z-stacks of 6 µm each. Image analyses were performed with ImageJ software (NIH, Bethesda, USA). To eliminate variations in size between cases, we calculated for each one the surface and the volume of the surface to be analyzed. Quantified numbers for NPFF-positive cells were normalized to the volume.

### Statistics

The number of NPFF-immunoreactive cells was counted for each region (gray matter, the white matter divided into compartments I-V, and deep white matter) and then divided by the volume of the region for each single case. The data were averaged and presented as a mean ± SEM.

Statistical comparisons between the different cortical areas in the superficial and compartments of the white matter and between the different groups of individuals were performed by using one-way ANOVA test with Dunnet’s multiple comparison test with *p < 0.05 to ****p < 0.0001. All statistical studies were performed with the GraphPad Prism 9.4.1 statistical package (GraphPad Software, San Diego, California, USA).

## Results

### Distribution and morphology of gNPFF and wNPFF cells in the normal human cerebral cortex of *n* = 9 individuals without neurological disease

First, we characterized the distribution of NPFF in 100 µm hemisphere sections from the following neocortical regions of controls: cingulate gyrus, frontal gyrus, superior temporal gyrus, and the medial and inferior temporal gyri (Suppl. Table 1 and Suppl. Figure 2a).

The anti-NPFF antibody labeled a morphologically heterogeneous population of medium-sized cells (gNPFF cells) distributed throughout the cerebral cortex and the underlying superficial white matter (wNPFF). The somata were uniformly and consistently stained and most lacked deposits of lipofuscin granules. Within the neocortical gray matter, NPFF cells were found in all superficial layers (I-IV); they increased numerically in infragranular layers V and VI. Most wNPFF-positive cells were located in compartments I and II at a depth of 1000 µm below the gray matter (Fig. 1a, b). As previously reported for WMIC [24, 41], wNPFF cells were most abundant in the gyral crowns of the neocortex (Fig. 1b), decreased along the flanks of the gyri, and were least abundant at the base of the sulci. However, NPFF cells were also found in the deep white matter (> 2500 µm under GM-WM border). Twenty percent of NPFF-immunoreactive cells were located in the cortical gray matter, 80% were localized in the white matter, of which 63% occurred in the superficial white matter and 17% in deep white matter.

**Fig. 2:**
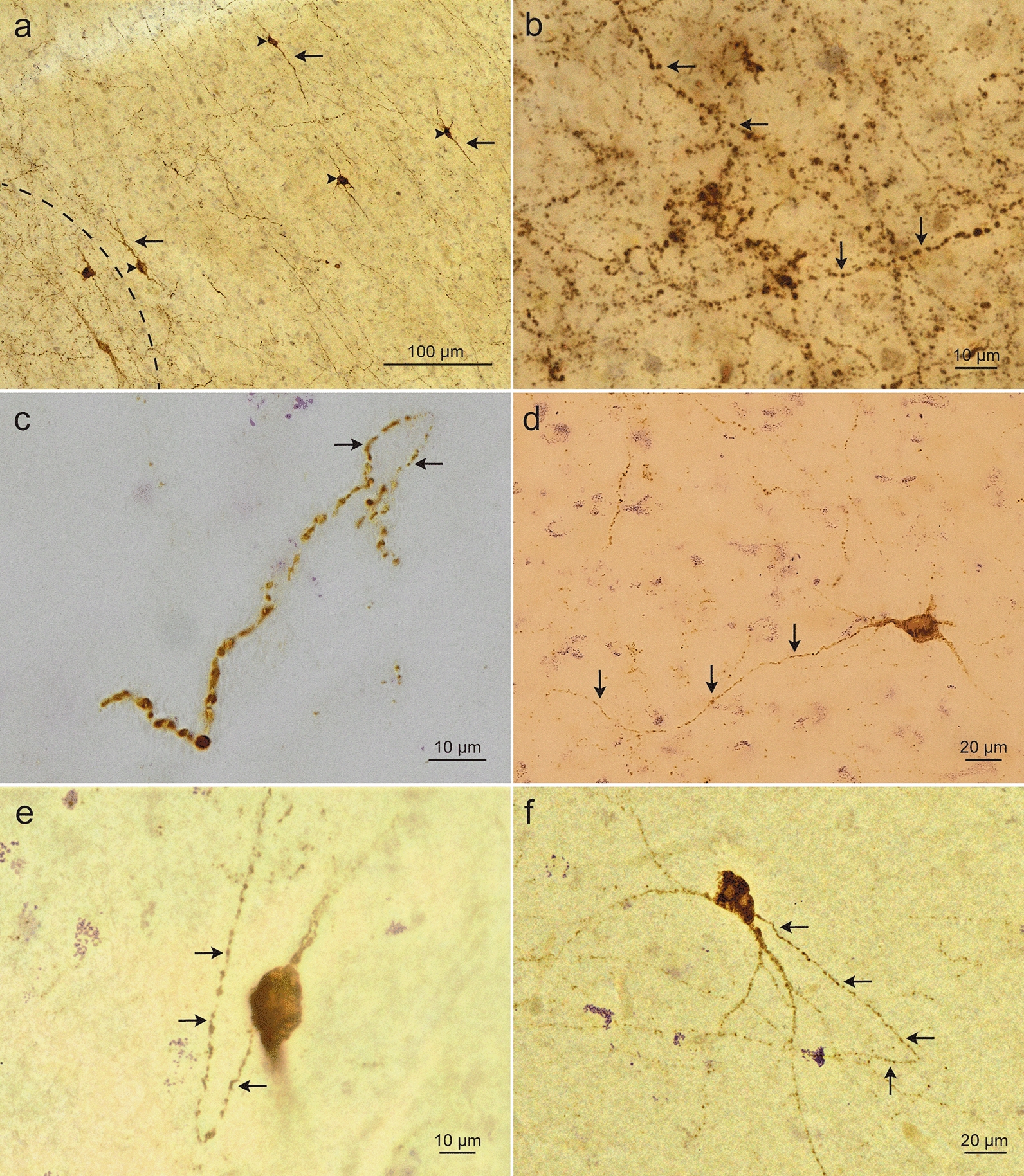
White and gray matter NPFF-immunopositive cellular processes in control cases. **a**. The cellular processes (axons) of white matter NPFF-positive cells *(arrowheads)* displayed bead-like varicosities *(arrows)* and ran chiefly parallel to the white matter/gray matter border (*broken line*), here in the superior temporal gyrus. Case 7: female, 79 years of age. Pigment (aldehyde fuchsin) staining plus NPFF-IHC (brown chromogen DAB). **b** A network of NPFF-positive cellular processes in the gray matter of the superior temporal gyrus. *Arrows* point to bead-like varicosities. Same individual and staining as in **a**. **c** Looped (*arrows*) NPFF-positive cellular processes (axons) with bead-like varicosities from the cingulate gyrus gray matter. Case 8: male, 83 years of age. Pigment (aldehyde fuchsin) staining plus NPFF-IHC. **d** Multipolar NPFF-immunoreactive cell in the superficial white matter of the superior temporal gyrus with a long and tapering dendrite (*arrows*) rather than an extensively branching dendritic arbor. Same case and staining profile as in **a** and **b**. **e, f** Examples of NPFF-positive cells with lengthy axons displaying bead-like varicosities (*arrows*) in the superficial white matter of the frontal gyrus (**e**, bipolar/ovoid, same case as in **c)** and of the striate area 17 (**f**, case 9: female, 87 years of age). Pigment (aldehyde fuchsin) staining plus NPFF-IHC. PEG-embedded sections of 100 µm thickness **(a–f)**

Cell forms could be distinguished based on the shapes of their perikarya. gNPFF cells with pyramidal-like (triangular) and multipolar somata predominated within the gyral crowns (Fig. 1c, d), whereas bipolar shapes as well as ovoid cells prevailed in the sulcal depths (Fig. 1e, f). gNPFF and wNPFF cells alike were elongated in shape, with a longitudinal axis of 20 ± 5 µm (gNPFF)/21 ± 4 µm (wNPFF) and an average somal width of 12 ± 3 µm (gNPFF)/14 ± 2 µm (wNPFF). At opposite poles, a few conically shaped, smoothly contoured, lengthy, and gradually tapering dendrites emerged that seldom gave off side-branches (Fig. 2d). The longitudinal axis of the somata of wNPPF cells was mostly oriented tangentially to the border between the cortical gray ribbon and immediately subjacent white matter (Fig. 2a). There, the axon-like cellular processes shared a distinctive feature: multiple varicosities resembling a string of beads spaced closely but evenly apart (Fig. 2b, e, f). In contrast, most of the gNPFF cells in cortical layers II and III displayed radially aligned cellular processes, running at right angles to the cortical surface, and sometimes forming loops (Fig. 2c). Very few NPFF-positive cellular processes with varicosities were located in the small-celled granular layer IV (thereby making it likely that these processes were not thalamocortical afferents), whereas the infragranular layers V-VI displayed them in greater numbers.

Notably, whereas axon-like cellular processes with NPFF-positive varicosities were also located in the superficial white matter association (U-fiber) zone of the cortical convolutions, in the deep white matter, including the large fiber bundles there (internal capsule, anterior commissure, corpus callosum, fornix) NPFF-positive processes with beaded varicosities were lacking. Otherwise, deep white matter NPFF-positive axon-like processes were also directed to the gyral surface and devoid of short lateral branches. All axon-like processes within the gray and white matter emanating from the NPFF-positive somata were negatively labeled for myelin and the axonal marker SMI-31. The NPFF antibody did not label oligodendrocytes or astrocytes.

### NPFF expression in the neocortex

**Fig. 3:**
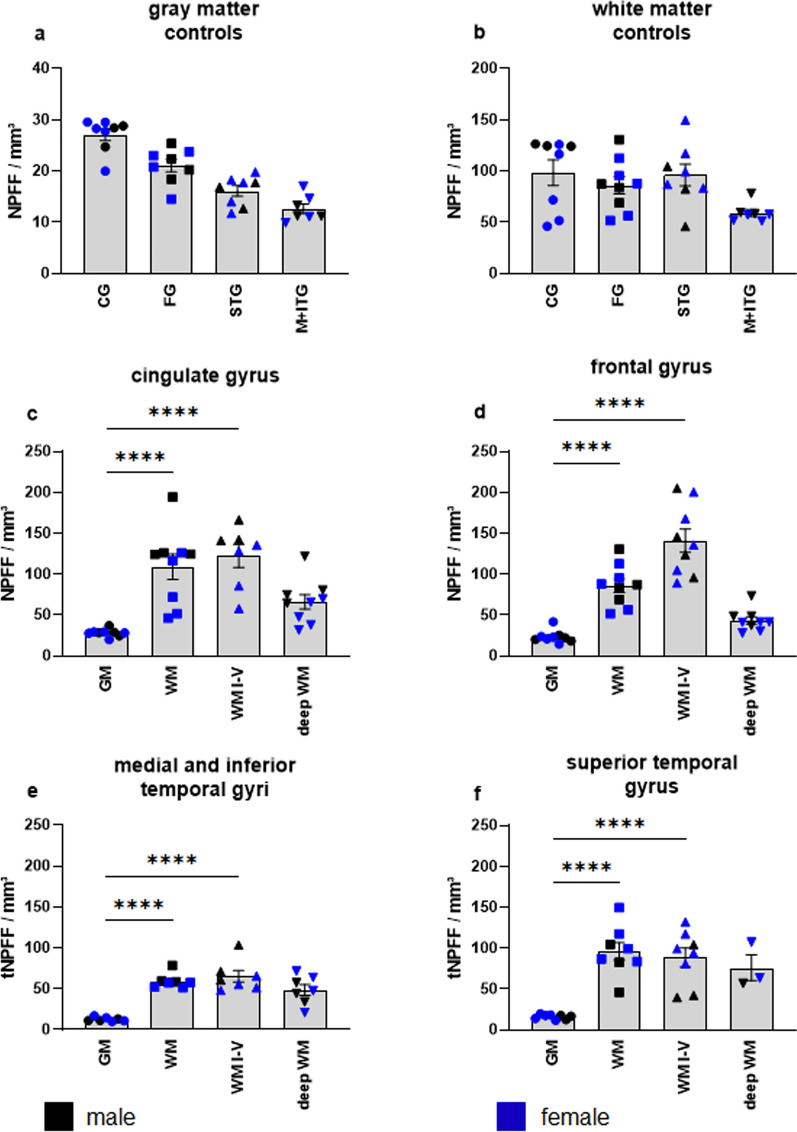
NPFF-immunopositive cells/mm^3^ in the neocortical gray and white matter of *n* = 9 control cases. **a**. Cell counts in the gray matter of the cingulate gyrus (CG), frontal gyrus (FG), superior temporal gyrus (STG), and medial plus inferior temporal gyri (M + ITG) in 4 males (*black*) and 5 females (*blue*). See also Supplementary Table 1. **b.** Cell counts in the white matter of the same regions and individuals as in **a**. **c.** Cell counts in 4 males (*black*) and 5 females (*blue*) in the gray matter (GM), white matter (WM), white matter compartments I-V (WM I-V), and deep white matter (deep WM) of the cingulate gyrus. **d.** Cell counts in the gray matter (GM), white matter (WM), white matter compartments I–V (WM I-V), and deep white matter (deep WM) of the frontal gyrus. **e.** Cell counts in the gray matter (GM), white matter (WM), white matter compartments I-V (WM I-V), and deep white matter (deep WM) of the medial and inferior gyri. **f.** Cell counts in the gray matter (GM), white matter (WM), white matter compartments I-V (WM I-V), and deep white matter (deep WM) of the superior temporal gyrus. Quantification of NPFF-positive cells was normalized to the volume of analyzed surface area. Data are presented as mean ± SEM and analyzed by one-way ANOVA followed by Dunnett’s multiple comparisons test. Statistically significant differences are indicated (****p < 0.0001)

Medium-sized gNPFF-cells were present in all of the regions studied. The number of gNPFF cells averaged 21 ± 7 NPFF/mm^3^, with the cingulate gyrus having the largest number of NPFF-positive cells. Their number tended to decrease approaching the temporal gyri (Table 1, Fig. 3a). By contrast, wNPFF were four times more numerous (Fig. 3b). The cingulate, frontal, und superior temporal gyri contained more gNPFF per surface area than the medial and inferior temporal gyri (Table 1, Fig. 3b). However, the differences did not reach significance. All of the regions displayed a comparable number of wNPFF-immunoreactive cells within the white matter compartments I-V (Fig. 3c–f). Numerous NPFF-positive cells were also found in the deep white matter compartments, accounting for up to 18% of the total number of wNPFF-positive cells (Fig. 3c–f).

**Table 1:** Stereological results for NPFF cell numbers in neocortical and allocortical gray and white matter of n = 9 control cases

NPFF / mm^3^		CG			FG			STG			M + ITG			ER			HF		
		*n*	mean	SEM	*n*	mean	SEM	*n*	mean	SEM	*n*	mean	SEM	*n*	mean	SEM	*n*	mean	SEM
GM	Ctrl	**9**	**29.68**	**0.6238**	**9**	**23.35**	**0.8447**	**9**	**18.16**	**0.7453**	**7**	**12.65**	**0.3570**	**6**	**19.12**	**0.7866**	**5**	**11.09**	**2.4057**
	male	4	32.33	1.4677	4	21.59	0.7507	4	20.46	2.4056	3	11.94	0.3952	3	16.78	1.0049	3	11.44	4.8948
	female	5	27.02	0.8013	5	24.76	2.0322	5	16.31	0.6632	4	13.19	0.8162	3	21.45	1.8345	2	10.57	6.0536
WM	Ctrl	**9**	**120.52**	**5.6536**	**9**	**86.09**	**2.8167**	**9**	**107.85**	**4.9794**	**7**	**59.24**	**1.2840**	**6**	**82.71**	**3.3912**	**5**	**48.47**	**7.7075**
	male	4	158.59	9.4106	4	92.69	6.6393	4	108.50	16.5364	3	65.40	3.7503	3	74.93	8.8773	3	50.67	15.0151
	female	5	82.45	7.3605	5	80.82	5.2079	5	107.34	5.4204	4	54.62	0.8197	3	90.49	4.0013	2	45.18	21.4843
WM I-V	Ctrl	**9**	**145.07**	**9.4573**	**9**	**140.95**	**4.7715**	**0**	**NE**	**–**	**7**	**65.16**	**2.6692**	**6**	**78.63**	**4.4234**	**0**	**NE**	**–**
	male	4	207.63	17.2807	4	142.60	11.6020	0	NE	**–**	3	78.49	7.4024	3	84.44	13.1177	0	NE	**–**
	female	5	82.50	10.7189	5	139.64	9.0954	0	NE	**–**	4	55.15	1.9076	3	72.83	3.5157	0	NE	**–**
																			**–**
deep WM	Ctrl	**9**	**74.65**	**3.6831**	**9**	**43.47**	**1.4738**	**0**	**NE**	**–**	**7**	**48.67**	**2.5019**	**4**	**93.94**	**13.8241**	**0**	**NE**	**–**
	male	4	98.63	7.3027	4	52.10	3.7941	0	NE	**–**	3	45.41	3.8987	2	46.13	2.6208	0	NE	**–**
	female	5	50.68	3.3587	5	36.56	1.3413	0	NE	**–**	4	51.12	5.6105	2	141.75	0.8237	0	NE	**–**

Bold indicate values for overall control group (male + female)

Quantitatively determined number of all NPFF-positive cells on average of the control cases examined normalized to the calculated volume of the areal analyzed (NPFF/mm^3^) for the following regions: cingulate gyrus (CG), frontal gyrus (FG), superior temporal gyrus (STG), medial and inferior temporal gyrus (M + ITG), entorhinal region (ER), and hippocampal formation (HF). Inclusive separation for males and females (male, female), number of cases analyzed (n), average of the determined number of NPFF-positive cells in the region analyzed (mean), control (Ctrl), gray matter (GM), white matter (WM), white matter compartments I-V (WM I-V), deep white matter (deep WM), standard deviation of mean (SEM), not evaluated (NE), SEM not calculable (–)

All regions had significantly more wNPFF-positive cells than gNPFF cells (Fig. 3c–f). In the cingulate gyrus, 30 ± 0.6 gNPFF/mm^3^ were found (52% of them in the infragranular layers V-VI) and 121 ± 6 wNPFF/mm^3^ (****p < 0.0001) (Table 1, Fig. 3c). The numerically largest portion in the white matter resided in compartment I (52%), followed by 14% in compartment II, but also up to 17% in the deep white matter. In the frontal gyrus, 23 ± 1 gNPFF/mm^3^ were found and 86 ± 3 wNPFF/mm^3^ (****p < 0.0001), whereby 82% of the wNPFF were located in compartments I-V and 18% in the deeper white matter layers (Table 1, Fig. 3d). A similar distribution pattern was observable in the temporal lobe, where we examined the temporal, medial, und inferior gyri, all of which displayed significantly greater numbers of wNPFF cells than gNPFF cells (Table 1, Fig. 3e, f). Our analyses revealed no gender-related differences, and scores for males and females were homogeneous, as were scores for younger and older individuals (Suppl. Figure 3).

### NPFF expression in the primary visual neocortex

For the two cases analyzed, the number of gNPFF cells in the striate area 17 averaged 9 ± 1 NPFF/mm^3^ compared to the cingulate gyrus with 30 ± 0.6 NPFF/mm^3^, the frontal gyrus with 23 ± 1 NPFF/mm^3^, and the superior temporal gyrus with 18 ± 1 NPFF/mm^3^. In both cases, 89.3% of NPFF-positive cells were observed in the superficial white matter of the primary visual neocortex. The distribution of wNPFF-positive cells in compartments I-V resembled closely that in the other neocortical regions examined, with the numerically largest portions in compartment I (62%), followed by 22% in compartment II, and decreasing in compartments III-V. The deep white matter aspect was too thin to be accurately evaluated.

The density of wNPFF-positive cells in the primary visual neocortex (compartments I-V) was far below the densities in the other regions examined: Whereas 145 NPFF/mm^3^ were found in the cingulate gyrus and 140 NPFF/mm^3^ in the frontal gyrus (for the superior temporal gyrus, where the boundary between compartments and deep white matter could not be reliably identified, the overall white matter wNPFF density was 108 NPFF/mm^3^), only 78 NPFF/mm^3^ were located in the striate area. Inasmuch as only two cases were studied, statistical comparison was not possible.

### NPFF expression in the allocortex

All morphological subtypes mentioned above for the neocortex were also found in the allocortex of all controls. Triangular and multipolar forms were observed most frequently compared to the bipolar and ovoid forms. NPFF cells occurred in outer entorhinal layers (except for layer pre-α), in the lamina dissecans, in deep layers and in the lamina cellularis profunda [6]. Within the hippocampal formation, gNPFF cells were sparsely distributed and located chiefly in the stratum oriens of sectors CA1 and CA2. Their numbers decreased in sectors CA3 and CA4. In exceptional instances, we also saw them in the dentate fascia. The number of gNPFF-positive cells was lower than in the neocortical areas (Table 1). In the lamina dissecans of the entorhinal cortex, the gNPFF cells formed in some instances a chain-like row (Suppl. Figure 2).

In the hippocampal formation, 1 mm^3^ of white matter (alveus) contained 49 ± 8 wNPFF; in the entorhinal region the number rose to 83 ± 3 wNPFF/mm^3^ (Table 1). More wNPFF were found in the entorhinal region than in the gray matter (entorhinal gray matter 19 ± 1 gNPFF/mm^3^) (Table 1). Gender-specific or age-specific effects were not detectable in these regions using the Pearson correlation test (data not shown).

Loop-like cellular processes with multiple bead-like varicosities were again detectable in the allocortex. As in the neocortex, these were SMI-31-negative and stained negatively for myelin. The entorhinal cortex displayed the same bead-like varicosities that we had observed in neocortical regions. Axons with NPFF-positive bead-like varicosities also existed in CA1-CA4, but the direction of their orientation was haphazard rather than uniform. A few NPFF-positive cellular processes were also seen in CA4 and in the dentate fascia but were lacking in the alveus and fornix.

### Somatostatin (SOM), calretinen (CR), calbindin (CB), parvalbumin (PV), and neuronal nitric oxide synthase (nNOS) expression

**Fig. 4:**
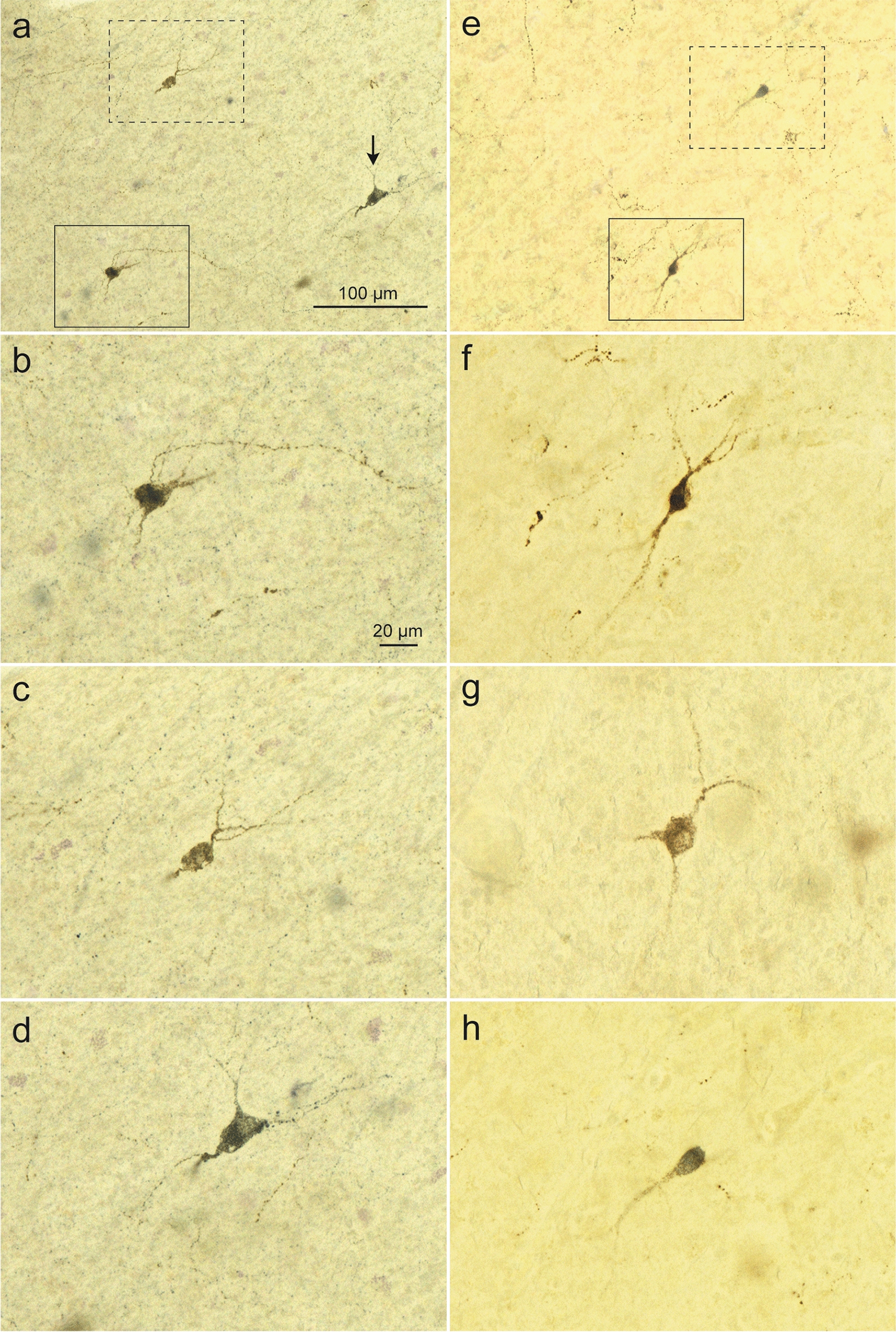
NPFF-somatostatin and NPFF-nNOS double immunostaining in the frontal gyrus. **a–d** NPFF (brown chromogen DAB) and somatostatin (blue chromogen, SK4700 Vector) double immunostaining. Overview in **a** showing three cells, each of which is shown at higher magnification in **b–d** NPFF/somatostatin-positive cell within *solid framed area* in **a** is shown at higher magnification in **b.** In** c** the cell is NPFF-positive (DAB) but negative for somatostatin (*dashed framed area* in **a**); in micrograph **d**, the cell shown (*arrow* in **a**) is immunopositive only for somatostatin (SK4700 Vector). Case 9: female, 87 years of age. **e–h** NPFF (brown chromogen DAB) and neuronal NOS (blue chromogen, SK4700 Vector) double immunostaining. Overview in **e** contains two nNOS immunopositive cells (*frames*) shown at higher magnification in **f** (NPFF/nNOS-positive) and **h** (immunopositive only for nNOS, SK4700 Vector). The cell in **g** from elsewhere in the same slide as **e** is NPFF-positive (DAB) but nNOS-negative. Case 8: male, 83 years of age. 100 µm PEG-embedded sections from frontal gyrus (**a-g**). Scale bar in **a** is valid for **e**; scale bar in **b** also applies to **c**, **d**, and **f–h**

In the neocortex, 69 ± 5% of NPFF-positive cells were SOM-positive (Fig. 4a–d). The differences between the gray matter (64%), superifical white matter (74%), and deep white matter (68%) were small. Comparable numbers were also found in the allocortex, where a large proportion of SOM-positive cells expressed the neuropeptide NPFF, but, even there, a smaller subgroup did not display positive double immunostaining. 75 ± 2% of the total NPFF-positive cells located within the superficial and deep white matter of the frontal gyrus were nNOS-positive (Fig. 4e–h); no double immunostaining was observed in the gray matter. Neuronal NOS (type I) has been implicated not only in modulating physiological functions, such as learning, memory, neurogenesis, and the central regulation of blood pressure [46, 67, 91], but also in contributing to neurogeneration [69, 81]. The fact that NPFF cells co-expressed nNOS aligns well with NPFF’s putative role in regulating central autonomic responses [28, 29], whereas we still do not know if nNOS cells staining positively for NPFF are reduced or altered in some manner in any of the disorders we sampled here. In double immunoreactions directed against the calcium-binding proteins CR, CB, and parvalbumin (PV), NPFF-positive cells did not express CR, CB, or PV in any of the regions investigated.

### gNPFF and wNPFF cells in the cerebral cortex of *n* = 22 patients with selected neurodegenerative diseases

A detailed summary, including age, gender, neuropathologic disease stages for each case can be found in Supplementary Table 1. As in the control cases above, we examined the frontal, cingulate, superior temporal, medial and inferior temporal gyri, as well as the temporal allocortex in hemisphere sections from all patients. Here, the morphological features of the NPFF-positive cells (i.e., cell contours, cell size, axon-like cellular processes with bead-like varicosities), in these individuals did not differ from those described above in normal brains.

**Fig. 5:**
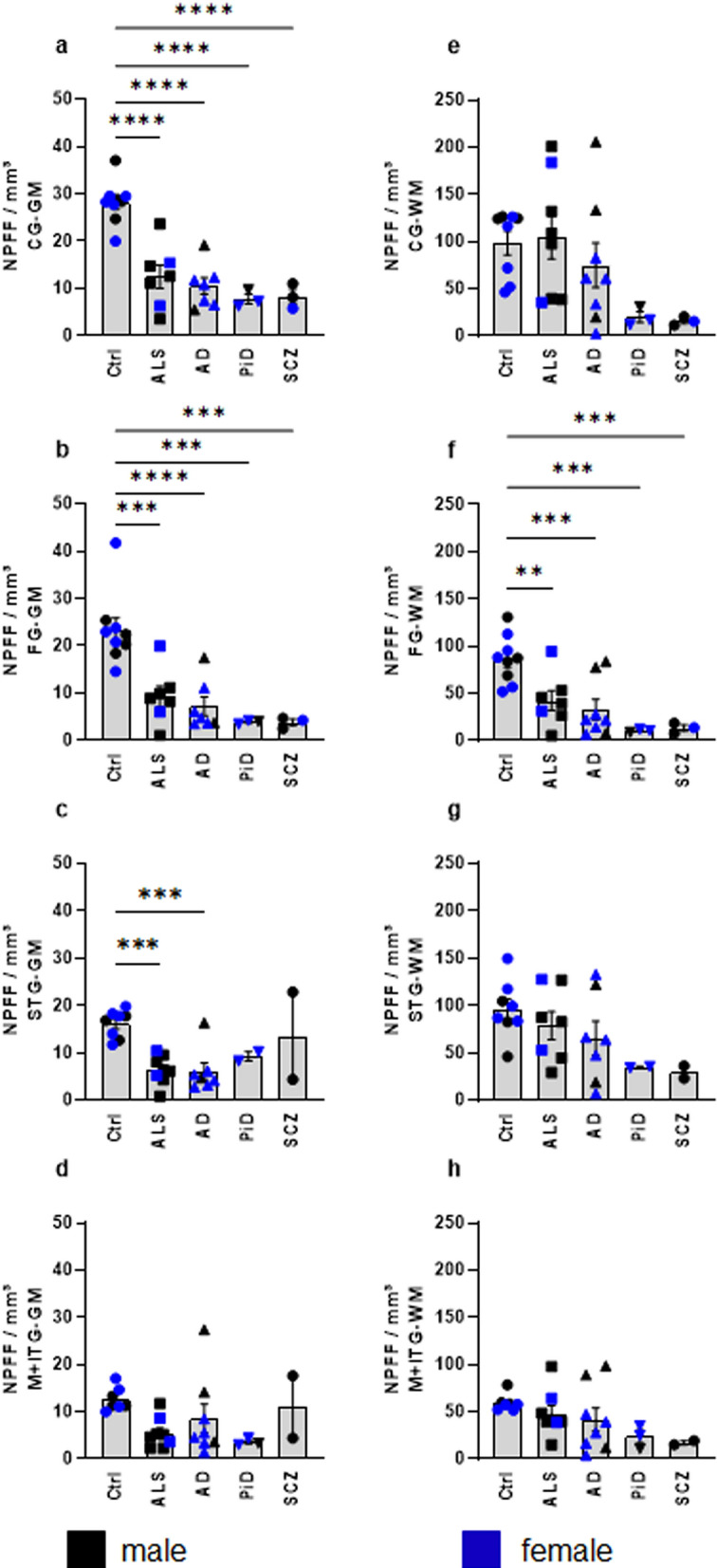
NPFF-immunopositive cells/mm^3^ in the neocortex of *n* = 9 control cases *versus* *n* = 22 patients with neurodegenerative disorders. **a**/**e** Cell counts in the gray/white matter of the cingulate gyrus (CG-GM, CG-WM). **b/f** Cell densities in the frontal gyrus (FG-GM, FG/WM), **c/g** superior temporal gyrus (STG-GM, STG-WM), and **d/h** medial plus inferior temporal gyri (M + ITG-GM, M + ITG-WM). Males (*black*) and females (*blue*). For the demographics and neuropathological diagnoses of the *n* = 9 controls (5 females, 4 males), *n* = 8 sporadic ALS patients (2 females, 6 males), *n* = 8 AD patients (5 females, 3 males), *n* = 3 PiD patients (2 females, 1 male), and *n* = 3 schizophrenia patients (1 female, 2 males), see Supplementary Table 1. Quantification of NPFF-positive cells was normalized to the volume of analyzed surface area. Data are presented as mean ± SEM and analyzed by one-way ANOVA followed by Dunnett ‘s multiple comparisons test. Statistically significant differences are indicated (*p < 0.05; **p < 0.005; ***p < 0.001; ****p < 0.0001)

**Fig. 6:**
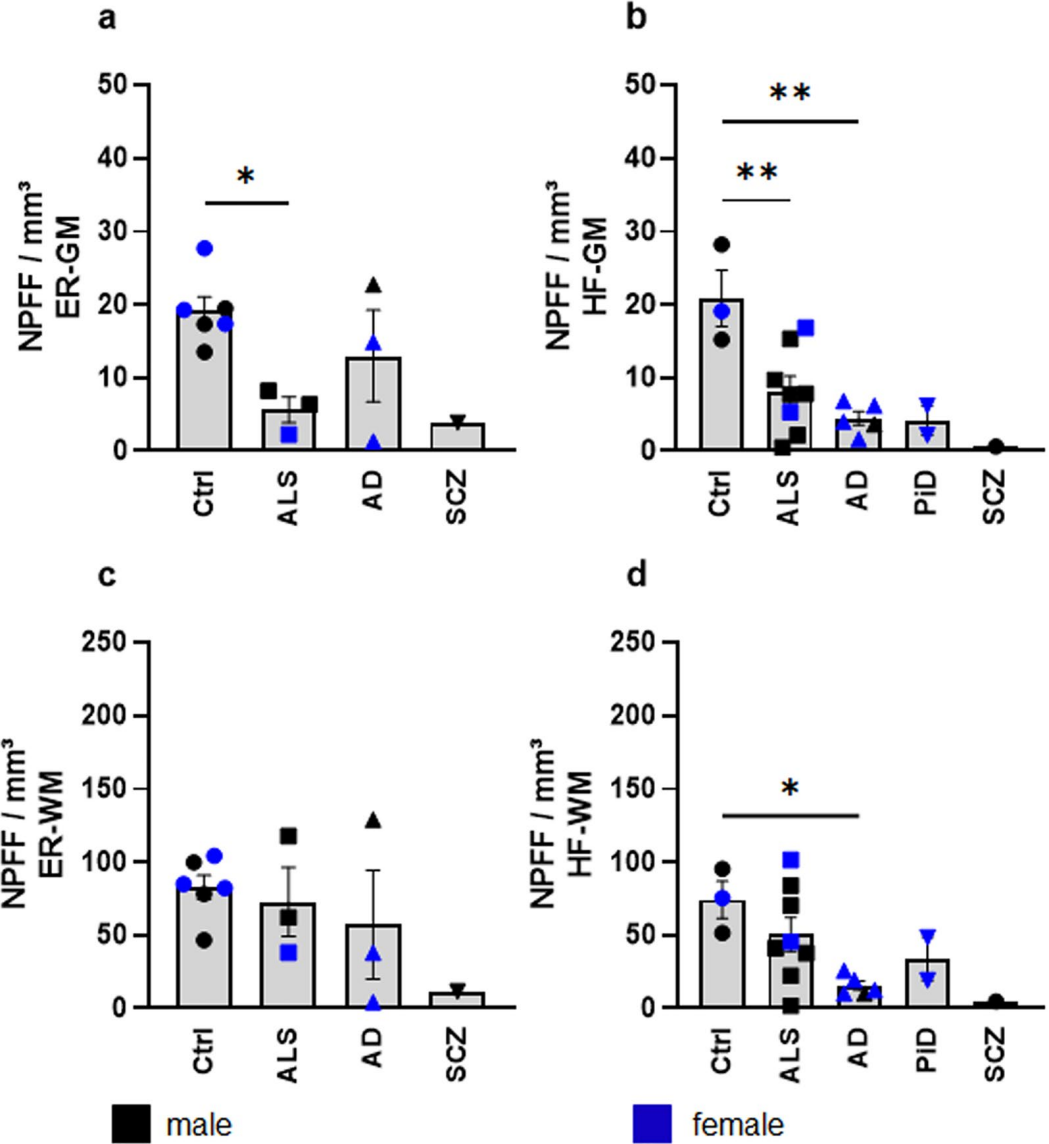
NPFF-immunopositive cells/mm^3^ in the allocortex of *n* = 9 control *versus* *n* = 22 patients with neurodegenerative disorders. **a/c** Cell counts in the gray/white matter of the entorhinal region (ER-GM, ER/WM) and **b/d** hippocampal formation (HF-GM, HF-WM). Males (*black*) and females (*blue*). For the demographics and neuropathological diagnoses of the controls (*n* = 6 ER, *n* = 5 HF), sporadic ALS patients (*n* = 3 ER, *n* = 8 HF), AD patients (*n* = 3 ER, *n* = 5 HF), PiD patients (*n* = 0 ER, *n* = 2 HF), and schizophrenia (*n* = 1 ER, *n* = 1 HF), see Supplementary Table 1. Quantification of NPFF-positive cells was normalized to the volume of analyzed surface area. Data are presented as mean ± SEM and analyzed by one-way ANOVA followed by Dunnett’s multiple comparisons test. Statistically significant differences are indicated (*p < 0.05 **p < 0.01). The hippocampal stratum oriens displayed only a few axon-like NPFF-positive cellular processes, whereas in the pyramidal cell layer of the subiculum and CA1-CA3 sectors a web-like NPFF-positive plexus existed. The stratum glomerulosum-moleculare and perforant path were marked by remarkably dense accumulations of axon-like NPFF-positive cellular processes

In sporadic ALS, we found significantly fewer gNPFF in the cingulate (****p < 0.0001), frontal (***p = 0.0003), and superior temporal gyri (***p = 0.0009) than in controls. There were no differences in the medial and inferior temporal gyri (p = 0.0672) compared to control brains (Fig. 5a–d). Notably, the gNPFF cell counts in the sole case with ALS + FTLD-TDP (Suppl. Table 1, case 14) were significantly lower in all regions than the ALS gray matter average cell counts for all regions (Supp. Table 3). Whereas in the superficial and deep white matter of the frontal gyrus, wNPFF cell counts were significantly lower (**p = 0.0083) (Fig. 5f) than those in controls, no difference was detected in the white matter of any of the remaining regions of the neocortex (Table 2, Fig. 5e–h). Nor was a significantly lower number of gNPFF-positive cells detectable in the entorhinal cortex of ALS patients in comparison to controls, although the cell counts in all layers (excluding layer pre-α) tended to be lower than in controls (Fig. 6a and c). The stratum oriens of sectors CA1 and CA2 of the hippocampal formation contained fewer gNPFF cells (**p = 0.0071) than control cases (Fig. 6b and d).

**Table 2:** Stereological results for NPFF-positive cells numbers in neocortical and allocortical gray and white matter of cases with sporadic amyotrophic lateral sclerosis, sporadic Alzheimer’s disease, Pick’s disease, and schizophrenia

NPFF/mm3		CG			FG			STG			M + ITG			ER			HF		
		*n*	Mean	SEM	*n*	Mean	SEM	*n*	Mean	SEM	*n*	Mean	SEM	*n*	Mean	SEM	*n*	Mean	SEM
GM	Ctrl	9	29.68	0.6238	9	23.35	0.8447	9	18.16	0.7453	7	12.65	0.3570	6	19.12	0.7866	5	11.09	2.4057
	Male	4	32.33	1.4677	4	21.59	0.7507	4	20.46	2.4056	3	11.94	0.3952	3	16.78	1.0049	3	11.44	4.8948
	Female	5	27.02	0.8013	5	24.76	2.0322	5	16.31	0.6632	4	13.19	0.8162	3	21.45	1.8345	2	10.57	6.0536
	ALS	8	16.43	1.5896	8	11.99	1.1603	8	6.39	0.3829	8	5.42	0.4077	3	5.62	1.0171	8	8.17	0.7196
	Male	6	18.29	2.3657	6	11.67	1.6723	6	5.89	0.5076	6	5.19	0.5797	2	7.29	0.6653	6	7.21	0.8922
	Female	2	10.84	3.2208	2	12.96	4.9306	2	7.88	1.8330	2	6.10	1.7869	1	2.27	–	2	11.07	4.0730
	AD	8	15.43	1.8171	8	9.94	1.1506	8	8.66	1.0507	8	8.54	1.0703	3	12.98	3.6186	7	8.94	1.2691
	Male	3	24.83	7.5572	3	16.81	4.2644	3	15.84	3.6409	3	15.02	3.9747	1	22.75	–	3	14.64	3.9858
	Female	5	9.79	0.5215	5	5.82	0.6273	5	4.35	0.2911	5	4.66	0.5274	2	8.10	4.8100	4	4.66	0.5920
	PiD	3	7.76	0.5924	3	3.89	0.1379	2	9.27	0.6811	3	3.58	0.2310	0	NE	–	2	4.19	1.4220
	Male	1	9.74	–	1	4.02	–	0	NE	–	1	3.33	–	0	NE	–	0	NE	–-
	Female	2	6.77	0.3341	2	3.83	0.2821	2	9.27	0.6811	2	3.71	0.4655	0	NE	–	2	4.19	1.4220
	Schizophrenia	3	8.31	0.8739	3	3.80	0.3913	2	13.60	6.5297	2	10.99	4.6653	1	3.88	–	1	0.61	–
	Male	2	8.40	1.8508	2	3.57	0.7847	2	13.60	6.5297	2	10.99	4.6653	1	3.88	–	1	0.61	–
	Female	1	8.14	–	1	4.24	–	0	NE	–	0	NE	–	0	NE	–	0	NE	–
WM	Ctrl	9	120.52	5.6536	9	86.09	2.8167	9	107.85	4.9794	7	59.24	1.2840	6	82.71	3.3912	5	48.47	7.7075
	Male	4	158.59	9.4106	4	92.69	6.6393	4	108.50	16.5364	3	65.40	3.7503	3	74.93	8.8773	3	50.67	15.0151
	Female	5	82.45	7.3605	5	80.82	5.2079	5	107.34	5.4204	4	54.62	0.8197	3	90.49	4.0013	2	45.18	21.4843
	ALS	8	104.55	8.1508	8	53.13	5.0501	8	95.43	7.4029	8	47.86	3.0314	3	72.75	13.6193	8	50.58	4.1088
	Male	6	102.86	10.1810	6	49.86	7.1373	6	97.13	10.9829	6	46.74	4.5719	2	89.98	19.7239	6	42.90	5.0491
	Female	2	109.62	52.5288	2	62.94	22.3569	2	90.34	26.3161	2	51.23	9.0021	1	38.27	–	2	73.61	19.7459
	AD	8	74.86	8.3134	8	32.66	3.8146	8	85.05	8.8228	8	41.68	4.3952	3	57.23	21.4882	7	38.90	6.0532
	Male	3	119.58	31.2339	3	56.37	14.0527	3	120.90	33.6926	3	66.37	15.8515	1	129.05	–	3	68.09	18.4903
	female	5	48.03	6.1686	5	18.43	1.6301	5	63.54	9.0935	5	26.86	3.4718	2	21.32	11.9853	4	17.01	1.7381
	PiD	3	19.85	3.2826	3	10.40	0.5639	2	34.95	0.4683	3	23.50	4.1023	0	NE	–	2	33.87	10.4721
	Male	1	30.84	–	1	8.63	–	0	NE	–	1	10.59	–	0	NE	–	0	NE	–
	Female	2	14.36	1.7931	2	11.29	0.5043	2	34.95	0.4683	2	29.96	3.6278	0	NE	–	2	33.87	10.4721
	Schizophrenia	3	15.70	1.4952	3	13.16	1.8231	2	29.75	4.6907	2	17.06	1.4535	1	11.55	–	1	4.76	–
	male	2	15.84	3.1672	2	12.91	3.8551	2	29.75	4.6907	2	17.06	1.4535	1	11.55	–	1	4.76	–
	Female	1	15.42	–	1	13.67	–	0	NE	–	0	NE	–	0	NE	–	0	NE	–
WM I-V	Ctrl	9	145.07	9.4573	9	140.95	4.7715	0	NE	–	7	65.16	2.6692	6	78.63	4.4234	0	NE	–
	Male	4	207.63	17.2807	4	142.60	11.6020	0	NE	–	3	78.49	7.4024	3	84.44	13.1177	0	NE	–
	Female	5	82.50	10.7189	5	139.64	9.0954	0	NE	–	4	55.15	1.9076	3	72.83	3.5157	0	NE	–
	als	8	147.69	10.6366	8	103.57	10.8626	0	NE	–	8	59.83	3.3789	3	73.08	14.0552	0	NE	–
	Male	6	150.83	13.6014	6	98.77	15.5656	0	NE	–	6	57.71	4.9370	2	90.48	20.8505	0	NE	–
	Female	2	138.26	65.4764	2	117.99	46.6270	0	NE	–	2	66.16	12.4537	1	38.27	–-	0	NE	–
	AD	8	87.18	10.4381	8	53.47	7.1312	0	NE	–	8	55.01	5.8663	3	60.69	20.7876	0	NE	–
	Male	3	143.38	38.5568	3	98.88	25.6893	0	NE	–	3	84.53	21.5221	1	127.71	–	0	NE	–
	Female	5	53.45	8.2994	5	26.23	3.1754	0	NE	–	5	37.29	5.3816	2	27.18	16.1296	0	NE	–
	PiD	3	21.76	3.7800	3	11.73	0.7170	0	NE	–	3	26.92	5.7487	0	NE	–	0	NE	–
	Male	1	34.69	–	1	10.52	–	0	NE	–	1	10.59	–	0	NE	–	0	NE	–
	Female	2	15.30	1.2843	2	12.33	1.3295	0	NE	–	2	35.08	6.9780	0	NE	–	0	NE	–
	Schizophrenia	3	12.75	0.4445	3	14.55	2.7936	0	NE	–	2	17.62	0.4603	1	8.14	–	0	NE	–
	Male	2	12.69	0.9406	2	15.07	5.8920	0	NE	–	2	17.62	0.4603	1	8.14	–	0	NE	–
	Female	1	12.85	–	1	13.51	–	0	NE	–	0	NE	–	0	NE	–	0	NE	–
deep WM	Ctrl	9	74.65	3.6831	9	43.47	1.4738	0	NE	–	7	48.67	2.5019	4	93.94	13.8241	0	NE	–
	Male	4	98.63	7.3027	4	52.10	3.7941	0	NE	–	3	45.41	3.8987	2	46.13	2.6208	0	NE	–
	female	5	50.68	3.3587	5	36.56	1.3413	0	NE	–	4	51.12	5.6105	2	141.75	0.8237	0	NE	–
	ALS	7	41.36	3.3296	8	16.60	1.2252	0	NE	–	7	21.54	0.5657	2	88.11	14.5263	0	NE	–
	Male	5	38.42	3.0615	6	17.34	1.9135	0	NE	–	5	22.40	0.4117	2	88.11	14.5263	0	NE	–
	Female	2	48.70	23.2984	2	14.37	0.1980	0	NE	–	2	19.38	4.0014	0	NE	–	0	NE	–
	AD	7	85.87	18.6838	8	11.68	0.6656	0	NE	–	8	16.63	1.4721	2	69.51	49.1514	0	NE	–
	Male	2	72.13	18.7312	3	12.17	2.4153	0	NE	–	3	21.56	1.4123	1	139.02	–	0	NE	–
	Female	5	91.36	31.7330	5	11.39	0.9607	0	NE	–	5	13.68	2.8609	1	0.00	–	0	NE	–
	PiD	3	7.55	2.6533	3	7.91	1.7935	0	NE	–	2	20.73	2.0436	0	NE	–	0	NE	–
	Male	1	6.78	–	1	4.91	–	0	NE	–	0	NE	–	0	NE	–	0	NE	–
	Female	2	7.93	5.6088	2	9.41	3.3305	0	NE	–	2	20.73	2.0436	0	NE	–	0	NE	–
	Schizophrenia	2	50.60	11.8979	3	12.40	1.0014	0	NE	–	2	15.97	3.8847	1	17.99	–	0	NE	–
	Male	1	67.42	–	2	11.70	1.9423	0	Ne	–	2	15.97	3.8847	1	17.99	–	0	NE	–
	Female	1	33.77	–	1	13.80	–	0	NE	–	0	NE	–	0	NE	–	0	NE	–

Quantitatively determined number of all NPFF-positive cells on average of the cases examined. Controls (Ctrl) and cases with neurodegenerative diseases (ALS—sporadic amyotrophic lateral sclerosis. AD—sporadic Alzheimer’s diesease. PiD—Pick’s disease. schizophrenia) were normalized to the calculated volume of the areal analyzed (NPFF/mm^3^) for the following regions: cingulate gyrus (CG), frontal gyrus (FG), superior temporal gyrus (STG), medial and inferior temporal gyrus (M + ITG), entorhinal region (ER), and hippocampal formation (HF). Inclusive separation for males and females (male, female). number of cases analyzed (n), average of the determined number of NPFF-positive cells in the region analyzed (mean), gray matter (GM), white matter (WM), white matter compartments I-IV (WM I-IV), deep white matter (deep WM), standard deviation of mean (SEM), not evaluated (NE), SEM not calculable (–)

AD brains (Suppl. Table 1, cases 18–25) displayed a NPFF regional distribution pattern resembling that seen in sporadic ALS (Table 2). gNPFF-positive cell counts were signifcantly lower than in controls in the cingulate (**** p < 0.0001), frontal (****p < 0.0001), and superior temporal gyri (***p = 0.0009) (Table 2, Fig. 5a–c). Furthermore, the quantity of wNPPF cells observed in the superficial and deep white matter of the frontal gyrus (***p = 0.0009) was significantly lower compared to controls (Table 2, Fig. 5e, f), whereas the white matter of the cingulate and superior temporal gyri did not display significantly fewer NPPF-positive cells. No differences existed between AD and control brains with regard to NPFF-positive cell counts in the medial and inferior temporal gyri (Table 2, Fig. 5d and h). We did not detect any significant difference (p = 0.2504) in mean deep white matter somal size in eight AD cases *versus* the control group. Significantly fewer gNPPF-positive cells were found in the hippocampal formation of late-stage AD cases (NFT stage V) as opposed to controls (**p = 0.0017, Fig. 6b and d), but the number of gNPFF cells counted in the entorhinal layers did not diverge significantly from that of controls.

In PiD (Suppl. Table 1, cases 26–28), the gNPFF cell counts reported for the cingulate an d frontal gyri were significantly lower (cingulate: ****p < 0.0001; frontal ***p = 0.0002; Fig. 5a, b) than in controls and, in addition, the numbers of wNPFF cells in these areals were also significantly lower (cingulate: p = 0.2046; frontal ***p = 0.0006) compared to wNPFF cell counts in controls (Fig. 5e, f). The regions of the superior, medial, and inferior temporal gyri also showed significantly fewer gNPFF and wNPFF cells than control cases.

In the brains of three schizophrenic patients (Suppl. Table 1, cases 29–31), we found significantly lower numbers of wNPFFs in the frontal gyrus (***p = 0.001) but not in cingulate, superior, or medial and inferior gyri (Table 2, Fig. 5e–h) in comparison to control cases. In the gray matter of the frontal and cingulate, but not temporal, gyri, a significantly lower number of gNPFF were detected (frontal ***p = 0.0002; cingulate ****p < 0.0001). For the single case for which both the entorhinal cortex and hippocampal formation were available, gNPFF cell counts were also lower than in controls.

### Absence of proteinopathy-associated aggregates in NPFF-positive neurons

In all of the brains studied, we looked to see whether the NPFF antibody co-localized with relevant disease-related proteinopathy markers. Here, NPFF-positive cells did not colocalize in double immunoreactions with inclusion body pathologies characteristic of sporadic amyotrophic lateral sclerosis (pTDP43) (Suppl. Table 1, cases 10–17) or synucleinopathies (**α**-synuclein) (Suppl. Table 1, cases 13 and 15). In twenty-four of 28 brains staged for AD-related pathology (Suppl. Table 1, cases 4–26, 28), the somata of WMIC that contained AT8-positive pretangles in the entorhinal cortex, as described previously [85], did not display NPFF-immunoreactivity. Extraneuronal β-amyloid plaques were also NPFF-negative (Suppl. Table 1, cases 6, 8, 15–25, 27, 28, 31); nor did we find NPFF-positive cytoplasmic Pick bodies or ballooned neurons in any of the PiD cases examined.

### Immunofluorescence (IF) results

We confirmed the neurochemical identity of the wNPFF by IF staining. More than 90% were immunopositive for VGAT (Suppl. Figure 4a–c), and less than 5% were positive for vGluT1/2. wNPFF inhibitory synapses displayed puncta on dendrites that were immunopositive both for the presynaptic marker VGAT and the post-synaptic inhibitory marker gephyrin (Suppl. Figure 4d–h). For a small number of cells, no immunoreactivity for excitatory or inhibitory markers could be detected.

## Discussion

Here, application of immunoreactions with an NPFF antibody to 100 µm sections of the human neocortex and allocortex permitted the first anatomical characterization of a hitherto unknown NPFF-positive population of neurons located in in superficial and deep aspects of the white matter and in the gray matter, showing that the majority are located in the superficial white matter and thereby also making NPFF a new marker for studying these heterogeneous and incompletely understood cells. Earlier, García-Marín et al. [24] reported that, in the superficial white matter of the adult human brain, NeuN-positive white matter neurons varied signifcantly depending on the cortical region examined. In our control cohort, the number of wNPFF densities did not fluctuate greatly from region to region. We observed the greatest number of superficial wNPFFs in the superior temporal (84.2%) gyrus, followed by the cingulate (83.4%) gyrus. A lower density was found in the frontal gyrus (81.7%) and, as seen by others [24, 54], the lowest densities existed in the striate area 17 in both instances where the primary visual neocortex was examined. In the deep white matter, where García-Marín et al. [24] found that the density of NeuN-positive white matter neurons was similar in all cortical areas examined, we also observed little variation in the density of NPFF-positive cells between the neocortical regions sampled. Similarly to the results of García-Marín for NeuN-positive WM neurons, there were approximately four times more wNPFF-positive neurons in the superficial than in the deep white matter of our controls [24].

In addition to mapping the distribution of NPFF-positive cells in human cerebral cortex, we exploited NPFF immunohistochemistry to characterize wNPFF and gNPFF cells in cases with neuropathologically confirmed sporadic ALS, AD, PiD, and schizophrenia. To distinguish our cells from the conventional WMIC, a term that presently applies exclusively to neurons located in the superficial white matter and includes a disparate population of both glutamatergic projection cells and GABAergic interneurons [56], we designated them as gray matter NPFF-positive cells (gNPPF) and, in the white matter, as NPFF-positive cells (wNPPF).

Up to now, there have been no reports of NPFF-positive WMIC in any species, although NPFF immunoreactivity has been reported in and outside of the CNS [25–27, 29, 48–50, 53, 62]. Studies of NPFF in humans have been limited almost exclusively to the hypothalamus [25–27], where, for example, Goncharuk et al. [27] reported the presence of NPFF-immunoreactive punctate varicosities in cellular processes of hypothalamic nuclei, a feature that we repeatedly encountered in all of the regions studied here. Neither that group nor we can explain the origins or functions or these axonal varicosities.

The morphological characteristics (pyramidal-like, bipolar, multipolar, long cellular processes) and locations of the NPFF-positive cells support the conclusion that they can be classified as medium-sized nerve cells, with a somal size of 20–21 μm. Their NPFF-positive cellular processes, which were predominantly radially aligned between pyramidal cells of the neocortex and allocortex, were located in the immediately subjacent white matter rather than in the deep white matter. Presumably, the cellular processes are axons originating from NPFF nerve cells (Fig. 2d). Because branchings were rare and the terminal ends failed to show any branching, these cells do not belong to the class of interneurons with short and profusely branching axons. The NPFF-positive axons did not lie within the long association bundles, commissural connections, or efferent fibers of the neocortex that project to the brainstem and spinal cord. Nor were they present in the hippocampal alveus and fornix. Therefore, they are not cortical efferent projections.

The NPFF-positive cells revealed additional features characteristic of interneurons (e.g., somatostatin positivity); but it should be pointed out that many neuronal types are known to express multiple or diverse neuropeptides. Cortical interneurons are further classified into two main categories: excitatory glutamatergic and inhibitory GABAergic cells [56] located in both the gray and the white matter. The densities of interneurons differ from region to region, as did those of the NPFF cells (Fig. 3). Nonetheless, apart from the fact that approximately 68% of the NPFF cells were somatostatin-positive and displayed the same morphologies as cortical interneurons (bipolar, multipolar), they were, in contrast to the findings reported by others for WMIC in non-human primates and humans, calretinin- as well as calbindin- and parvalbumin-negative [20, 24, 75–78, 83, 94]. Thus, based on their morphology and topographical distribution, we conclude that the NPFF-positive neurons are neither classical short-axoned interneurons nor long-axoned projection neurons.

The presence of VGAT/gephyrin-immunoreactive puncta on NPFF-positive dendrites suggests that the wNPFF are integrated in synaptic networks within the U-fibers; however, because synaptic structures can be incompletely preserved in formalin-fixed tissues, thereby complicating the attribution of pre- and post-synaptic sites to specific cells, it was not possible to determine whether these were inhibitory synapses projecting to wNPFF or inhibitory synapses originating from the wNPFF neurons themselves and projecting to local targets. Nevertheless, these findings are in agreement with the reported local synaptic architectures detected in WMIC and their sensitivity to GABAergic inputs [84, 87].

Double-immunofluorescence enabled us to identify the wNPFF cells as inhibitory neurons. In light of the previously reported split into 50% excitatory and 50% inhibitory white matter neurons [24], our finding implies that wNPFF cells represent a fraction of the total population of WMIC in the human cerebral cortex. In agreement with this model, the overall density of wNPFF cells was substantially lower than the density of NeuN-positive white matter cells reported by the same group. Furthermore, the neurochemical characterization of the wNPFF cells (only 6% of NeuN-positive were NOS-positive in [24] compared to 75% NOS-positive wNPFF cells here) is also compatible with the idea that wNPFF cells are a subset of the WMIC population.

Our data in controls and in cases with neurodegenerative disorders did not show significant age-dependent neuronal cell loss in the superficial and in deep white matter population of NPFF-positive cells. This finding resembles the situation described previously by Mortazavi et al. [57] for rhesus monkeys, with the exception of a moderate decrease in frontal region deep white matter neurons. Subtle age-dependent morphological changes in white matter neurons could have multiple causes given the wide range of putative white matter neuronal functions and their involvement in both corticothalamic and corticocortical circuits [57].

We did not utilize the term ‘neuronal loss’ to describe the significantly lower numbers of NPFF-positive cells in the brains of patients with sporadic ALS and selected tauopathies or in schizophrenia compared to controls because we found no evidence of NPFF-positive cell death (e.g., indicated by the presence of activated microglia, extraneuronal lipofuscin remnants) in pigment-Nissl stained hemisphere sections. However, the lower or reduced NPFF-positive cell counts could point to an underfunction of the NPFF network in such individuals, possibly contributing to deficient or dysfunctional interactions of NPFF with other neurotransmitter systems and cellular networks.

As a rule, most of the superficial wNPFF cells and their distinctively ‘beaded’ axons were concentrated in a zone that harbors the U-fibers (fibrae arcuatae cortici) in humans and monkeys, rather than in the deep white matter. Significant exceptions were the external and extreme capsules, where NPFF-positive axons were abundant. By contrast, other large white matter fiber bundles, including the uncinate fascicle, cingulum, superior and inferior longitudinal fascicles, occipitofrontal, and arcuate fascicles, were devoid of them. Not found in rodents, the U-fibers are local, short-range, glutamatergic corticocortical association fibers that connect adjacent cortical areas [11, 30, 58, 59, 90]. Previous studies indicate that not only specific subsets of WMIC are pathologically altered in humans, such as autism spectrum disorder and schizophrenia, but also that the U-fiber system is affected in AD [65], autism [19, 74], schizophrenia, and bipolar disorder [31, 64]. The function or possible interaction between NPFF-positive nerve cells and the U-fiber systems in all of these disorders is, to date, completely unknown and requires additional investigation.

Both their proximity to the corticocortical U-fibers and the presence of NPFF-positive axo-axonic inhibitory synapses (Suppl. Figure 4) distributed in the corresponding superficial white matter suggest an involvement in the gating of short-range (local) corticocortical circuits (but see also 84). U-fiber functioning is highly modulated during cognitive tasks [38], and therefore the degree of intracortical connectivity may be dependent on wNPFF (and WMIC) to ensure high signal-to-noise transmission. Thus, one could speculate that the superficial wNPFF-positive neurons in the human brain provide metabolic support or ‘fine tuning’ (modulation) to the U-fiber system. Under this assumption, loss or reduction of wNPFF in the U-fiber zone may substantially reduce the efficiency of intracortical connectivity and lead to the functional isolation of cortical areas in neurodegenerative and neurodevelopmental disorders.

In conclusion, we can now add the neuropeptide NPFF to the pool of markers available for studying the distribution, morphology, and neurochemistry of WMIC, which are characterized by enormous neurochemical heterogeneity: glutamatergic, but mostly GABAergic predominate, but there are also subpopulations displaying acetylcholinesterase, nicotinamide-adenine dinucleotide phosphate diaphorase (NADPH), MAP2, somatostatin, calbindin-D28K, calretinin, and parvalbumin [13, 21, 24, 33, 41, 42, 45, 47, 54, 75, 94]. The observed density and the partial overlap between somatostatin-positive and nNOS-positive WMIC (but not between calretinin-positive or calbindin-positive WMIC) and wNPFF cells suggests that NPFF-positive neurons could overlap with previously identified WMIC subpopulations. As such, NPFF can serve as an immunohistochemical marker useful for the investigation of WMIC neurons in the normal brain as well as in neurodegenerative and neurodevelopmental disorders. In addition, NPFF immunohistochemistry opens up novel perspectives for future applications in neuroanatomical and neuropathological studies of the wNPFF and gNPFF neurons in the human brain.

### Supplementary Information

Supplementary Material 1.

Supplementary Material 2.

Supplementary Material 3.

Supplementary Material 4.

Supplementary Material 5.

Supplementary Material 6.

Supplementary Material 7.

## References

[1] Akbarian S, Bunney WE, Potkin SG, Wigal SB, Hagman JO, Sandman CA, Jones EG (1993). Altered distribution of nicotinamide-adenine dinucleotide phosphatediaphorase cells in frontal lobe of schizophrenics implies disturbances of cortical development. Arch Gen Psychiatry.

[2] Akbarian S, Viñuela A, Kim JJ, Potkin SG, Bunney WE, Jones EG (1993). Distorted distribution of nicotinamide-adenine dinucleotide phosphatediaphorase neurons in temporal lobe of schizophrenics implies anomalous cortical development. Arch Gen Psychiatry.

[3] Akbarian S, Kim JJ, Potkin SG, Hetrick WP, Bunney WE, Jones EG (1996). Maldistribution of interstitial neurons in prefrontal white matter of the brains of schizophrenic patients. Arch Gen Psychiatry.

[4] Bailey A, Palferman S, Heavey L, Le Couteur A (1998). Autism: the phenotype in relatives. J Autism Dev Disord.

[5] Braak H, Braak E (1991). Demonstration of amyloid deposits and neurofibrillary changes in whole brain sections. Brain Pathol.

[6] Braak H, Braak E (1992). The human entorhinal cortex: normal morphology and lamina-specific pathology in various diseases. Neurosci Res.

[7] Braak H, Del Tredici K, Rüb U, de Vos RA, Jansen Steur EN, Braak E (2003). Staging of brain pathology related to sporadic Parkinson’s disease. Neurobiol Aging.

[8] Braak H, Thal DR, Ghebremedhin E, Del Tredici K (2011). Stages of the pathologic process in Alzheimer disease: age categories from 1 to 100 years. J Neuropathol Exp Neurol.

[9] Braak H, Ludolph AC, Neumann M, Ravits J, Del Tredici K (2017). Pathological TDP-43 changes in Betz cells differ from those in bulbar and spinal α- motoneurons in sporadic amyotrophic lateral sclerosis. Acta Neuropathol.

[10] Brettschneider J, Del Tredici K, Toledo JB, Robinson JL, Irwin DJ, Grossman M, Suh E, Van Deerlin VM, Wood EM, Baek Y, Kwong L, Lee EB, Elman L, McCluskey L, Fang L, Feldengut S, Ludolph AC, Lee VM, Braak H, Trojanowski JQ (2013). Stages of pTDP-43 pathology in amyotrophic lateral sclerosis. Ann Neurol.

[11] Catani M, Dell’acqua F, Vergani F, Malik F, Hodge H, Roy P, Valabregue R, De Schotten MT (2012). Short frontal lobe connections of the human brain. Cortex.

[12] Choudhury P, Scharf EL, Paolini MA, Graff-Radford J, Alden EC, Machulda MM, Jones DT, Fields JA, Murray ME, Graff-Radford NR, Constantopoulos E, Reichard RR, Knopman DS, Duffy JR, Dickson DW, Parisi JE, Josephs KA, Petersen RC, Boeve BF (2020). Pick’s disease: clinicopathologic characterization of 21 cases. J Neurol.

[13] Chun JJ, Shatz CJ (1989). Interstitial cells of the adult neocortical white matter are the remnant of the early generated subplate neuron population. J Comp Neurol.

[14] Clancy B, Silva-Filho M, Friedlander MJ (2001). Structure and projections of white matter neurons in the postnatal rat visual cortex. J Comp Neurol.

[15] Colombo JA (2018). Cellular complexity in subcortical white matter: a distributed control circuit?. Brain Struct Funct.

[16] Conner CM, Guo Y, Akbarian S (2009). Cingulate white matter neurons in schizophrenia and bipolar disorder. Biol Psychiatry.

[17] Connor CM, Crawford BC, Akbarian S (2011). White matter neuron alterations in schizophrenia and related disorders. Int J Dev Neurosci.

[18] Dai Y, Zhao X, Chen P, Yu Y, Wang Y, Xie L (2015). Neuropeptide FF promotes recovery of corneal nerve injury associated with hyperglycemia. Invest Ophthalmol Vis Sci.

[19] d’Albis MA, Guevara P, Guevara M, Laidi C, Boisgontier J, Sarrazin S, Duclap D, Delorme R, Bolognani F, Czech C, Bouquet C, Ly-Le Moal M, Holiga S, Amestoy A, Scheid I, Gaman A, Leboyer M, Poupon C, Mangin JF, Houenou J (2018). Local structural connectivity is associated with social cognition in autism spectrum disorder. Brain.

[20] DeFelipe J, Douglas Fields R, Hof PR, Höistad M, Kostović I, Meyer G, Rockland KS (2010). Cortical white matter: beyond the pale remarks, main conclusion and discussion. Front Neuroanat.

[21] Duchatel RJ, Shannon Weickert C, Tooney PA (2019). White matter neuron biology and neuropathology in schizophrenia. NPJ Schizophr.

[22] Eastwood SL, Harrison PJ (2003). Interstitial white matter neurons express less reelin and are abnormally distributed in schizophrenia: towards an integration of molecular and morphologic aspects of the neurodevelopmental hypothesis. Mol Psychiatry.

[23] Eastwood SL, Harrison PJ (2005). Interstitial white matter neuron density in the dorsolateral prefrontal cortex and parahippocampal gyrus in schizophrenia. Schizophr Res.

[24] García-Marín V, Blazquez-Llorca L, Rodriguez JR, Gonzalez-Soriano J, DeFelipe J (2010). Differential distribution of neurons in the gyral white matter of the human cerebral cortex. J Comp Neurol.

[25] Goncharuk VD, Buijs RM, Mactavish D, Jhamandas JH (2006). Neuropeptide FF distribution in the human and rat forebrain: a comparative immunohistochemical study. J Comp Neurol.

[26] Goncharuk VD, Buijs RM, Jhamandas JH, Swaab DF (2011). Vasopressin (VP) and neuropeptide FF (NPFF) systems in the normal and hypertensive human brainstem. J Comp Neurol.

[27] Goncharuk VD, Buijs RM, Jhamandas JH, Swaab DF (2014). The hypothalamic neuropeptide FF network is impaired in hypertensive patients. Brain Behav.

[28] Gouardères C, Puget A, Zajac JM (2004). Detailed distribution of neuropeptide FF receptors (NPFF1 and NPFF2) in the rat, mouse, *Octodon*, rabbit, *Guinea* pig, and *Marmoset* monkey brains: a comparative autoradiographic study. Synapse.

[29] Gutierrez-Mecinas M, Bell A, Polgár E, Watanabe M, Todd AJ (2019). Expression of neuropeptide FF defines a population of excitatory interneurons in the superficial dorsal horn of the mouse spinal cord that respond to noxious and pruritic stimuli. Neuroscience.

[30] Hutchinson EB, Schwerin SC, Radomski KL, Sadeghi N, Jenkins J, Komlosh ME, Irfanoglu MO, Juliano SL, Pierpaoli C (2017). Population based MRI and DTI templates of the adult ferret brain and tools for voxelwise analysis. Neuroimage.

[31] Ji E, Guevara P, Guevara M, Grigis A, Labra N, Sarrazin S, Hamdani N, Bellivier F, Delavest M, Leboyer M, Tamouza R, Poupon C, Mangin JF, Houenou J (2019). Increased and decreased superficial white matter structural connectivity in schizophrenia and bipolar disorder. Schizophr Bull.

[32] Joshi D, Fung SJ, Rothwell A, Weickert CS (2012). Higher gamma-aminobutyric acid neuron density in the white matter of orbital frontal cortex in schizophrenia. Biol Psychiatry.

[33] Judaš M, Sestan N, Kostović I (1999). Nitrinergic neurons in the developing and adult human telencephalon: transient and permanent patterns of expression in comparison to other mammals. Microsc Res Tech.

[34] Judaš M, Sedmak G, Pletikos M (2010). Early history of subplate and interstitial neurons: from Theodor Meynert (1867) to the discovery of the subplate zone (1974). J Anat.

[35] Judaš M, Sedmak G, Pletikos M, Jovanov-Milošević N (2010). Populations of subplate and interstitial neurons in fetal and adult human telencephalon. J Anat.

[36] Kilduff TS, Cauli B, Gerashchenko D (2011). Activation of cortical interneurons during sleep: an anatomical link to homeostatic sleep regulation?. Trends Neurosci.

[37] Kirkpatrick B, Messias NC, Conley RR, Roberts RC (2003). Interstitial cells of the white matter in the dorsolateral prefrontal cortex in deficit and nondeficit schizophrenia. J Nerv Ment Dis.

[38] Kitazawa Y, Sonoda M, Sakakura K, Mitsuhashi T, Firestone E, Ueda R, Kambara T, Iwaki H, Luat AF, Marupudi NI, Sood S, Asano E (2023). Intra- and inter-hemispheric network dynamics supporting object recognition and speech production. Neuroimage.

[39] Kivipelto L, Majane EA, Yang HY, Panula P (1989). Immunohistochemical distribution and partial characterization of FLFQPQRFamidelike peptides in the central nervous system of rats. J Comp Neurol.

[40] Kivipelto L, Panula P (1991). Origin and distribution of neuropeptide-FF-like immunoreactivity in the spinal cord of rats. J Comp Neurol.

[41] Kostović I, Rakic P (1980). Cytology and time of origin of interstitial neurons in the white matter in infant and adult human and monkey telencephalon. J Neurocytol.

[42] Kostović I, Stefulj-Fucić A, Mrzljak L, Jukić S, Delalle I (1991). Prenatal and perinatal development of the somatostatin-immunoreactive neurons in the human prefrontal cortex. Neurosci Lett.

[43] Kostović I, Judaš M, Sedmak G (2011). Developmental history of the subplate zone, subplate neurons and interstitial white matter neurons: relevance for schizophrenia. Int J Dev Neurosci.

[44] Kotani M, Mollereau C, Detheux M, Le Poul E, Brézillon S, Vakili J, Mazarguil H, Vassart G, Zajac JM, Parmentier M (2001). Functional characterization of a human receptor for neuropeptide FF and related peptides. Br J Pharmacol.

[45] Kowall NW, Beal MF (1988). Cortical somatostatin, neuropeptide Y, and NADPH diaphorase neurons: normal anatomy and alterations in Alzheimer’s disease. Ann Neurol.

[46] Krukoff TL (1999). Central actions of nitric oxide in regulation of autonomic functions. Brain Res Rev.

[47] Kubo KI (2020). Increased densities of white matter neurons as a cross-disease feature of neuropsychiatric disorders. Psychiatry Clin Neurosci.

[48] Lin YT, Chen JC (2019). Neuropeptide FF modulates neuroendocrine and energy homeostasis through hypothalamic signaling. Chin J Physiol.

[49] Lin YT, Yu Z, Tsai SC, Hsu PH, Chen JC (2020). Neuropeptide FF receptor 2 inhibits capsaicin-induced CGRP Upregulation in mouse trigeminal ganglion. J Headache Pain.

[50] Lin Z, Wang Y, Lin S, Liu D, Mo G, Zhang H, Dou Y (2021). Identification of potential biomarkers for abdominal pain in IBS patients by bioinformatics approach. BMC Gastroenterol.

[51] Mackenzie IR, Neumann M, Baborie A, Sampathu DM, Du Plessis D, Jaros E, Perry RH, Trojanowski JQ, Mann DM, Lee VM (2011). A harmonized classification system for FTLD-TDP pathology. Acta Neuropathol.

[52] Mai JK, Paxinos G, Voss T (2008). Atlas of the human brain.

[53] Majane EA, Yang HY (1987). Distribution and characterization of two putative endogenous opioid antagonist peptides in bovine brain. Peptides.

[54] Meyer G, Wahle P, Castaneyra-Perdomo A, Ferres-Torres R (1992). Morphology of neurons in the white matter of the adult human neocortex. Exp Brain Res.

[55] Meynert T (1867). Der Bau der Grosshirnrinde und seine örtlichen Verschiedenheiten, nebst einem pathologischanatomischen corollarium. Vierteljschr Psychiat.

[56] Molnár Z, Clowry GJ, Šestan N, Alzu'bi A, Bakken T, Hevner RF, Hüppi PS, Kostović I, Rakic P, Anton ES, Edwards D, Garcez P, Hoerder-Suabedissen A, Kriegstein A (2019). New insights into the development of the human cerebral cortex. J Anat.

[57] Mortazavi F, Wang X, Rosene DL, Rockland KS (2016). White matter neurons in young adult and aged rhesus monkey. Front Neuroanat.

[58] Nieuwenhuys R, Voogd J, van Huijzen C, Nieuwenhuys R, Voogd J, van Huijzen C (2008). Association and commissural connections. The human centrals nervous system.

[59] Oishi K, Zilles K, Amunts K, Faria A, Jiang H, Li X, Akhter K, Hua K, Woods R, Toga AW (2008). Human brain white matter atlas: identification and assignment of common anatomical structures in superficial white matter. Neuroimage.

[60] Okhotin VE, Kupriyanov VV (1997). Neurovascular relationships in the human neocortex. Neurosci Behav Physiol.

[61] Okhotin VE, Kalinichenko SG (2003). Subcortical white matter interstitial cells: their connections, neurochemical specialization, and role in the histogenesis of the cortex. Neurosci Behav Physiol.

[62] Panula P, Aarnisalo AA, Wasowicz K (1996). Neuropeptide FF, a mammalian neuropeptide with multiple functions. Prog Neurobiol.

[63] Perry SJ, Yi-Kung Huang E, Cronk D, Bagust J, Sharma R, Walker RJ, Wilson S, Burke JF (1997). A human gene encoding morphine modulating peptides related to NPFF and FMRFamide. FEBS Lett.

[64] Phillips OR, Nuechterlein KH, Asarnow RF, Clark KA, Cabeen R, Yang Y (2011). Mapping corticocortical structural integrity in schizophrenia and effects of genetic liability. Biol Psychiatry.

[65] Phillips OR, Joshi SH, Piras F, Orfei MD, Iorio M, Narr KL, Shattuck DW, Caltagirone C, Spalletta G, Di Paola M (2016). The superficial white matter in Alzheimer’s disease. Hum Brain Mapp.

[66] Phillips OR, Joshi SH, Squitieri F, Sanchez-Castaneda C, Narr K, Shattuck DW, Caltagirone C, Sabatini U, Di Paola M (2016). Major superficial white matter abnormalities in Huntington’s disease. Front Neurosci.

[67] Qadri F, Arens T, Schwarz EC, Häuser W, Dendorfer A, Dominiak P (2003). Brain nitric oxide synthase activity in spontaneously hypertensive rats during the development of hypertension. J Hypertens.

[68] Quillet R, Ayachi S, Bihel F, Elhabazi K, Ilien B, Simonin F (2016). RF-amide neuropeptides and their receptors in Mammals: pharmacological properties, drug development and main physiological functions. Pharmacol Ther.

[69] Reif A, Grünblatt E, Herterich S, Wichart I, Rainer MK, Jungwirth S, Danielczyk W, Deckert J, Tragl KH, Riederer P, Fischer P (2011). Association of a functional NOS1 promoter repeat with Alzheimer’s disease in the VITA cohort. J Alzheimers Dis.

[70] Richter Z, Janszky J, Sétáló G, Horváth R, Horváth Z, Dóczi T, Seress L, Ábrahám H (2016). Characterization of neurons in the cortical white matter in human temporal lobe epilepsy. Neuroscience.

[71] Sandvik GK, Hodne K, Haug TM, Okubo K, Weltzien FA (2014). RFamide peptides in early vertebrate development. Front Endocrinol.

[72] Sedmak G, Judaš M (2019). The total number of white matter interstitial neurons in the human brain. J Anat.

[73] Sedmak G, Judaš M (2021). White matter interstitial neurons in the adult human brain: 3% of cortical neurons in quest for recognition. Cells.

[74] Shukla DK, Keehn B, Smylie DM, Muller RA (2011). Microstructural abnormalities of short-distance white matter tracts in autism spectrum disorder. Neuropsychologia.

[75] Suarez-Sola ML, Gonzalez-Delgado FJ, Pueyo-Morlans M, Medina-Bolivar OC, Hernandez-Acosta NC, Gonzalez-Gomez M, Meyer G (2009). Neurons in thE white matter of the adult human neocortex. Front Neuroanat.

[76] Swiegers J, Bhagwandin A, Sherwood CC, Bertelsen MF, Maseko BC, Hemingway J, Rockland KS, Molnár Z, Manger PR (2019). The distribution, number, and certain neurochemical identities of infracortical white matter neurons in a lar gibbon (*Hylobates lar*) brain. J Comp Neurol.

[77] Swiegers J, Bhagwandin A, Williams VM, Maseko BC, Sherwood CC, Hård T, Bertelsen MF, Rockland KS, Molnár Z, Manger PR (2021). The distribution, number, and certain neurochemical identities of infracortical white matter neurons in a chimpanzee (*Pan troglodytes*) brain. J Comp Neurol.

[78] Swiegers J, Bhagwandin A, Maseko BC, Sherwood CC, Hård T, Bertelsen MF, Spocter MA, Molnár Z, Manger PR (2021). The distribution, number, and certain neurochemical identities of infracortical white matter neurons in the brains of a southern lesser galago, a black-capped squirrel monkey, and a crested macaque. J Comp Neurol.

[79] Tan RH, Shepherd CE, Kril JJ, McCann H, McGeachie A, McGinley C, Affleck A, Halliday GM (2013). Classification of FTLD-TDP cases into pathological subtypes using antibodies against phosphorylated and non-phosphorylated TDP43. Acta Neuropathol Commun.

[80] Thom M, Sisodiya S, Harkness W, Scaravilli F (2001). Microdysgenesis in temporal lobe epilepsy. A quantitative and immunohistochemical study of white matter neurones. Brain.

[81] Thorns V, Hansen L, Masliah E (1988). nNOS expressing neurons in the entorhinal cortex and hippocampus are affected in patients with Alzheimer’s disease. Exp Neurol.

[82] Tolnay M, Clavaguera F (2004). Argyrophilic grain disease: a late-onset dementia with distinctive features among tauopathies. Neuropathology.

[83] Tomioka R, Rockland KS (2007). Long-distance corticocortical GABAergic neurons in the adult monkey white and gray matter. J Comp Neurol.

[84] Torres-Reveron J, Friedlander MJ (2007). Properties of persistent postnatal cortical subplate neurons. J Neurosci.

[85] van de Nes JAP, Sandmann-Keil D, Braak H (2002). Interstitial cells subjacent to the entorhinal region expressing somatostatin-28 immunoreactivity are susceptible to development of Alzheimer’s disease-related cytoskeletal changes. Acta Neuropathol.

[86] von Engelhardt J, Khrulev S, Eliava M, Wahlster S, Monyer H (2011). 5-HT(3A) receptor-bearing white matter interstitial GABAergic interneurons are functionally integrated into cortical and subcortical networks. J Neurosci.

[87] Wahle P, Meyer G, Wu JY, Albus K (1987). Morphology and axon terminal pattern of glutamate decarboxylase-immunoreactive cell types in the white matter of the cat occipital cortex during early postnatal development. Brain Res.

[88] Yang HY, Fratta W, Majane EA, Costa E (1985). Isolation, sequencing, synthesis, and pharmacological characterization of two brain neuropeptides that modulate the action of morphine. Proc Natl Acad Sci USA.

[89] Yang Y, Fung SJ, Rothwell A, Tianmei S, Weickert CS (2011). Increased interstitial white matter neuron density in the dorsolateral prefrontal cortex of people with schizophrenia. Biol Psychiatry.

[90] Yoshino M, Saito K, Kawasaki K, Horiike T, Shinmyo Y, Kawasaki H (2020). The origin and development of subcortical U-fibers in gyrencephalic ferrets. Mol Brain.

[91] Zhou L, Zhu DY (2009). Neuronal nitric oxide synthase: structure, subcellular localization, regulation, and clinical implications. Nitric Oxide.

[92] Zhu H, Peng B, Klausen C, Yi Y, Li Y, Xiong S, von Dadelszen P, Leung PCK (2020). NPFF increases fusogenic proteins syncytin 1 and syncytin 2 via GCM1 in first trimester primary human cytotrophoblast cells. FASEB J.

[93] Zhukareva V, Shah K, Uryu K, Braak H, Del Tredici K, Sundarraj S, Clark C, Trojanowski JQ, Lee VM (2002). Biochemical analysis of tau proteins in argyrophilic grain disease, Alzheimer’s disease, and Pick’s disease : a comparative study. Am J Pathol.

[94] Zouridakis A, Ayala I, Minogue G, Kawles A, Keszycki R, Macomber A, Bigio EH, Geula C, Mesulam MM, Gefen T (2023). Shades of gray in human white matter. J Comp Neurol.

