# Supplementary material for: Neuropeptide FF (NPFF)-positive nerve cells of the human cerebral cortex and white matter in controls, selected neurodegenerative diseases, and schizophrenia
Source: Acta Neuropathol Commun. 2024 Jun 28;12:108. doi: 10.1186/s40478-024-01792-1 (PMC11212262; doi:10.1186/s40478-024-01792-1)
Supplement: Supplementary file 1 — Supplementary Material 1. [file 40478_2024_1792_MOESM1_ESM.docx]

**Supplementary Table 1. Demographics and clinicopathological data of the N = 31 cases studied.**

| **case** | **f/m** | **age** | **NFT** | **Aβ** | **α-syn** | **AGD** | **pTDP-43** | **clinical remarks** |
| --- | --- | --- | --- | --- | --- | --- | --- | --- |
|  |  |  |  |  |  |  |  |  |
| **1** | **♀** | 16 | 0 | 0 | 0 | 0 | 0 | aspiration pneumonia |
| **2** | **♂** | 26 | 0 | 0 | 0 | 0 | 0 | gunshot wound |
| **3** | **♀** | 44 | 0 | 0 | 0 | 0 | 0 | metastatic uterine cancer |
| **4** | **♂** | 46 | I | 0 | 0 | 0 | 0 | multisystem failure |
| **5** | **♀** | 55 | I | 0 | 0 | 0 | 0 | cause of death unknown |
| **6** | **♂** | 72 | II | 1 | 0 | 0 | 0 | sarcoma |
| **7** | **♀** | 79 | I | 0 | 0 | 0 | 0 | aortic dissection |
| **8** | **♂** | 83 | II | 2 | 0 | 0 | 0 | aspiration pneumonia |
| **9** | **♀** | 87 | II | 0 | 0 | 0 | 0 | cardiac failure |
|  |  |  |  |  |  |  |  |  |
| **10** | **♂** | 36 | I | 0 | 0 | 0 | 2 | ALS |
| **11** | **♀** | 43 | II | 0 | 0 | 0 | 3 | ALS |
| **12** | **♂** | 53 | I | 0 | 0 | 0 | 2 | ALS |
| **13** | **♂** | 56 | I | 0 | 2 | 0 | 2 | ALS + ILBD |
| **14** | **♂** | 68 | I | 0 | 0 | 0 | 4 | ALS + FTLD-TDP |
| **15** | **♂** | 75 | II | 1 | 3 | 0 | 2 | ALS + ILBD |
| **16** | **♀** | 76 | III | 1 | 0 | 0 | 2 | ALS |
| **17** | **♂** | 82 | III | 3 | 0 | 0 | 4 | ALS |
|  |  |  |  |  |  |  |  |  |
| **18** | **♀** | 61 | V | 3 | 0 | 0 | 0 | AD |
| **19** | **♀** | 74 | V | 4 | 0 | 0 | 0 | AD |
| **20** | **♀** | 76 | V | 3 | 0 | 0 | 0 | AD |
| **21** | **♀** | 83 | V | 5 | 0 | 0 | 0 | AD |
| **22** | **♂** | 83 | V | 4 | 0 | 0 | 0 | AD |
| **23** | **♂** | 85 | V | 3 | 0 | 0 | 0 | AD |
| **24** | **♂** | 88 | V | 3 | 0 | 0 | 0 | AD |
| **25** | **♀** | 92 | V | 5 | 0 | 0 | 0 | AD |
|  |  |  |  |  |  |  |  |  |
| **26** | **♀** | 70 | I | 0 | 0 | 0 | 0 | PiD |
| **27** | **♂** | 72 | 0 | 1 | 0 | 0 | 0 | PiD |
| **28** | **♀** | 74 | II | 3 | 0 | 0 | 0 | PiD |
|  |  |  |  |  |  |  |  |  |
| **29** | **♂** | 53 | I | 0 | 0 | 0 | 0 | schizophrenia |
| **30** | **♀** | 67 | II | 0 | 0 | 0 | 0 | schizophrenia |
| **31** | **♂** | 70 | I | 1 | 0 | 0 | 0 | schizophrenia |

**Abbreviations:** **f/m** – female/male; **age** – age in years; **NFT** – Alzheimer’s disease neurofibrillary stages 0-VI (AT8-immunohistochemistry, IHC) [8]; **Aβ** – amyloid-β deposition phase 0-5 (4G8-IHC [8]; **α-syn** – Parkinson’s disease neuropathological stages 0-6 (syn-1-IHC [7]; **AGD** – argyrophilic grain disease (AT8-IHC) [83, 94]; **pTDP-43** – sporadic amyotrophic lateral sclerosis neuropathological stages 0-4 (phosphorylated TDP-43-IHC) [10]; **ALS** – sporadic amyotrophic lateral sclerosis; **FTLD-TDP** – frontotemporal lobar degeneration with TDP pathology [51]; **ILBD** – incidental Lewy body disease; **PiD** – Pick’s disease (AT8-IHC [12]).
