# Supplementary material for: Neuropeptide FF (NPFF)-positive nerve cells of the human cerebral cortex and white matter in controls, selected neurodegenerative diseases, and schizophrenia
Source: Acta Neuropathol Commun. 2024 Jun 28;12:108. doi: 10.1186/s40478-024-01792-1 (PMC11212262; doi:10.1186/s40478-024-01792-1)
Supplement: Supplementary file 2 — Supplementary Material 2. [file 40478_2024_1792_MOESM2_ESM.docx]

**Supplementary Table 2. List of primary antibodies used for immunohistochemistry and immunofluorescence.**

| **1** | polyclonal antibody against the neuropeptide FF (anti-NPFF; 1:800; ab10352, Abcam, Cambridge, UK); *incubation 72 h |
| --- | --- |
|  |  |
| **2** | polyclonal rabbit antibody TDP-43 (1:2500; 10782-2-AP; Proteintech, Planegg-Martinsried, Germany) used as a neuronal marker to identify normal nuclear TDP-43 in the overall population of mature neurons [9, 80] |
|  |  |
| **3** | monoclonal antibody against phosphorylated tau (PHF-Tau; 1:2000; Clone AT8; Pierce Biotechnology, Rockford, IL, USA [Thermo Scientific]) used to detect Alzheimer-associated neurofibrillary pathology, Pick bodies, coiled bodies, thorn-shaped astrocytes, argyrophilic grains |
|  |  |
| **4** | monoclonal antibody anti-beta-amyloid (1:5000; Clone 4G8; BioLegend, San Diego, CA, USA) used to mark Aβ deposition |
|  |  |
| **5** | monoclonal antibody against α-synuclein (anti-syn-1; 1:2000; Clone number 42, BD Biosciences, Mountain View, CA, USA) used to visualize Lewy bodies/neurites and glial cytoplasmic inclusions |
|  |  |
| **6** | polyclonal rabbit antibody against phosphorylated TDP-43 (anti-phospho TDP-43 [pS409/410-2]; 1:5000; Cosmo Bio Co., Ltd., Tokyo, Japan) used for recognition of pTDP-43 cytoplasmic inclusions |
|  |  |
| **7** | monoclonal anti-neurofilament H (NF-H) phosphorylated antibody SMI-31 (1:2000; BioLegend, San Diego, CA, USA) used as an axonal marker |
|  |  |
| **8** | monoclonal myelin basic protein antibody (MBP; 1:1000; ab209328, Abcam, Cambridge, UK) |
|  |  |
| **9** | recombinant rabbit monoclonal antibody against somatostatin (Somatostatin; 1:1000; MA5-42760; Clone 7Q4T0; Invitrogen, Waltham, MA, USA [Thermo Scientific]); **citrate buffer pretreatment 30 min |
|  |  |
| **10** | polyclonal rabbit antibody against calretinin (Calretinin; 1:1000; PA5-34688; Invitrogen, Waltham, MA, USA [Thermo Scientific]); ** |
|  |  |
| **11** | monoclonal anti-parvalbumin (Parvalbumin; 1:1000, Clone: PARV-19; P3088; Sigma-Aldrich, Taufkirchen, Germany [Merck]); ** |
|  |  |
| **12** | polyclonal VGAT cytoplasmic domain (SYSY; 1:200; 131004; Göttingen, Germany) |
|  |  |
| **13** | monoclonal gephyrin antibody (SYSY; 1:500; 147009; Göttingen, Germany) |
