# Supplementary material for: Neuropeptide FF (NPFF)-positive nerve cells of the human cerebral cortex and white matter in controls, selected neurodegenerative diseases, and schizophrenia
Source: Acta Neuropathol Commun. 2024 Jun 28;12:108. doi: 10.1186/s40478-024-01792-1 (PMC11212262; doi:10.1186/s40478-024-01792-1)
Supplement: Supplementary file 3 — Supplementary Material 3. [file 40478_2024_1792_MOESM3_ESM.docx]

| **NPFF/mm³** | | **CG** | | |  | **FG** | | |  | **STG** | | |  | **M+ITG** | | |  | **ER** | | |  | **HF** | | |
| --- | --- | --- | --- | --- | --- | --- | --- | --- | --- | --- | --- | --- | --- | --- | --- | --- | --- | --- | --- | --- | --- | --- | --- | --- |
| **GM** | ALS | 8 | **16.43** | 1.5896 |  | 8 | **11.99** | 1.1603 |  | 8 | **6.39** | 0.3829 |  | 8 | **5.42** | 0.4077 |  | 3 | **5.62** | 1.0171 |  | 8 | **8.17** | 0.7196 |
|  | male | 6 | **18.29** | 2.3657 |  | 6 | **11.67** | 1.6723 |  | 6 | **5.89** | 0.5076 |  | 6 | **5.19** | 0.5797 |  | 2 | **7.29** | 0.6653 |  | 6 | **7.21** | 0.8922 |
|  | female | 2 | **10.84** | 3.2208 |  | 2 | **12.96** | 4.9306 |  | 2 | **7.88** | 1.8330 |  | 2 | **6.10** | 1.7869 |  | 1 | **2.27** | --- |  | 2 | **11.07** | 4.0730 |
|  | ALS + FTLD-TDP | 1 | **3.54** | --- |  | 1 | **1.05** | --- |  | 1 | **0.83** | --- |  | 1 | **2.22** | --- |  | 0 | **NE** | --- |  | 1 | **0.49** | --- |
|  |  |  |  |  |  |  |  |  |  |  |  |  |  |  |  |  |  |  |  |  |  |  |  |  |
| **WM** | ALS | 8 | **104.55** | 8.1508 |  | 8 | **53.13** | 5.0501 |  | 8 | **95.43** | 7.4029 |  | 8 | **47.86** | 3.0314 |  | 3 | **72.75** | 13.6193 |  | 8 | **50.58** | 4.1088 |
|  | male | 6 | **102.86** | 10.1810 |  | 6 | **49.86** | 7.1373 |  | 6 | **97.13** | 10.9829 |  | 6 | **46.74** | 4.5719 |  | 2 | **89.98** | 19.7239 |  | 6 | **42.90** | 5.0491 |
|  | female | 2 | **109.62** | 52.5288 |  | 2 | **62.94** | 22.3569 |  | 2 | **90.34** | 26.3161 |  | 2 | **51.23** | 9.0021 |  | 1 | **38.27** | --- |  | 2 | **73.61** | 19.7459 |
|  | ALS + FTLD-TDP | 1 | **38.59** | --- |  | 1 | **4.90** | --- |  | 1 | **29.39** | --- |  | 1 | **14.91** | --- |  | 0 | **NE** | --- |  | 1 | **1.73** | --- |
|  |  |  |  |  |  |  |  |  |  |  |  |  |  |  |  |  |  |  |  |  |  |  |  |  |
| **WM I-V** | ALS | 8 | **147.69** | 10.6366 |  | 8 | **103.57** | 10.8626 |  | 0 | **NE** | --- |  | 8 | **59.83** | 3.3789 |  | 3 | **73.08** | 14.0552 |  | 0 | **NE** | --- |
|  | male | 6 | **150.83** | 13.6014 |  | 6 | **98.77** | 15.5656 |  | 0 | **NE** | --- |  | 6 | **57.71** | 4.9370 |  | 2 | **90.48** | 20.8505 |  | 0 | **NE** | --- |
|  | female | 2 | **138.26** | 65.4764 |  | 2 | **117.99** | 46.6270 |  | 0 | **NE** | --- |  | 2 | **66.16** | 12.4537 |  | 1 | **38.27** | --- |  | 0 | **NE** | --- |
|  | ALS + FTLD-TDP | 1 | **38.59** | --- |  | 1 | **5.11** | --- |  | 0 | **NE** | --- |  | 1 | **14.91** |  |  | 0 | **NE** | --- |  | 0 | **NE** | --- |
|  |  |  |  |  |  |  |  |  |  |  |  |  |  |  |  |  |  |  |  |  |  |  |  |  |
| **deep WM** | ALS | 7 | **41.36** | 3.3296 |  | 8 | **16.60** | 1.2252 |  | 0 | **NE** | --- |  | 7 | **21.54** | 0.5657 |  | 2 | **88.11** | 14.5263 |  | 0 | **NE** | --- |
|  | male | 5 | **38.42** | 3.0615 |  | 6 | **17.34** | 1.9135 |  | 0 | **NE** | --- |  | 5 | **22.40** | 0.4117 |  | 2 | **88.11** | 14.5263 |  | 0 | **NE** | --- |
|  | female | 2 | **48.70** | 23.2984 |  | 2 | **14.37** | 0.1980 |  | 0 | **NE** | --- |  | 2 | **19.38** | 4.0014 |  | 0 | **NE** | --- |  | 0 | **NE** | --- |
|  | ALS + FTLD-TDP | 1 | **NE** | --- |  | 1 | **4.41** | --- |  | 0 | **NE** | --- |  | 0 | **NE** |  |  | 0 | **NE** | --- |  | 0 | **NE** | --- |

**Supplementary Table 3. Stereological results for NPFF-positive cells numbers in the neocortical gray and white matter of n = 8 cases with sporadic amyotrophic lateral sclerosis and comparison of group data with an ALS + FTLD-TDP case.**

Quantitatively determined number of all NPFF-positive cells on average of 8 ALS-cases examined and comparision of ALS group-data to case 14 (ALS + FTLD-TDP, in Suppl. Table 1) in *green*. Notably, the gray matter cell counts in the sole case with ALS + FTLD-TDP were substantially lower in all regions than the ALS gray matter average for all regions. Cases were normalized to the calculated volume of the areal analyzed (NPFF/mm³) for the following regions: cingulate gyrus (**CG**), frontal gyrus (**FG**), superior temporal gyrus (**STG**), medial (**MTG**) and inferior (**ITG**) temporal gyri, entorhinal region (**ER**), and hippocampal formation (**HF**). Inclusive separation for males and females (male. female), number of cases analyzed (**n**), average of the determined number of NPFF-positive cells in the region analyzed (**mean**), gray matter (**GM**), white matter (**WM**), white matter compartments I-V (**WM I-V**), deep white matter (**deep WM**), standard deviation of mean (**SEM**), not evaluated (**NE**), SEM not calculable (**---**).
