# Supplementary figures and images for: Neuropeptide FF (NPFF)-positive nerve cells of the human cerebral cortex and white matter in controls, selected neurodegenerative diseases, and schizophrenia
Source: Acta Neuropathol Commun. 2024 Jun 28;12:108. doi: 10.1186/s40478-024-01792-1 (PMC11212262; doi:10.1186/s40478-024-01792-1)

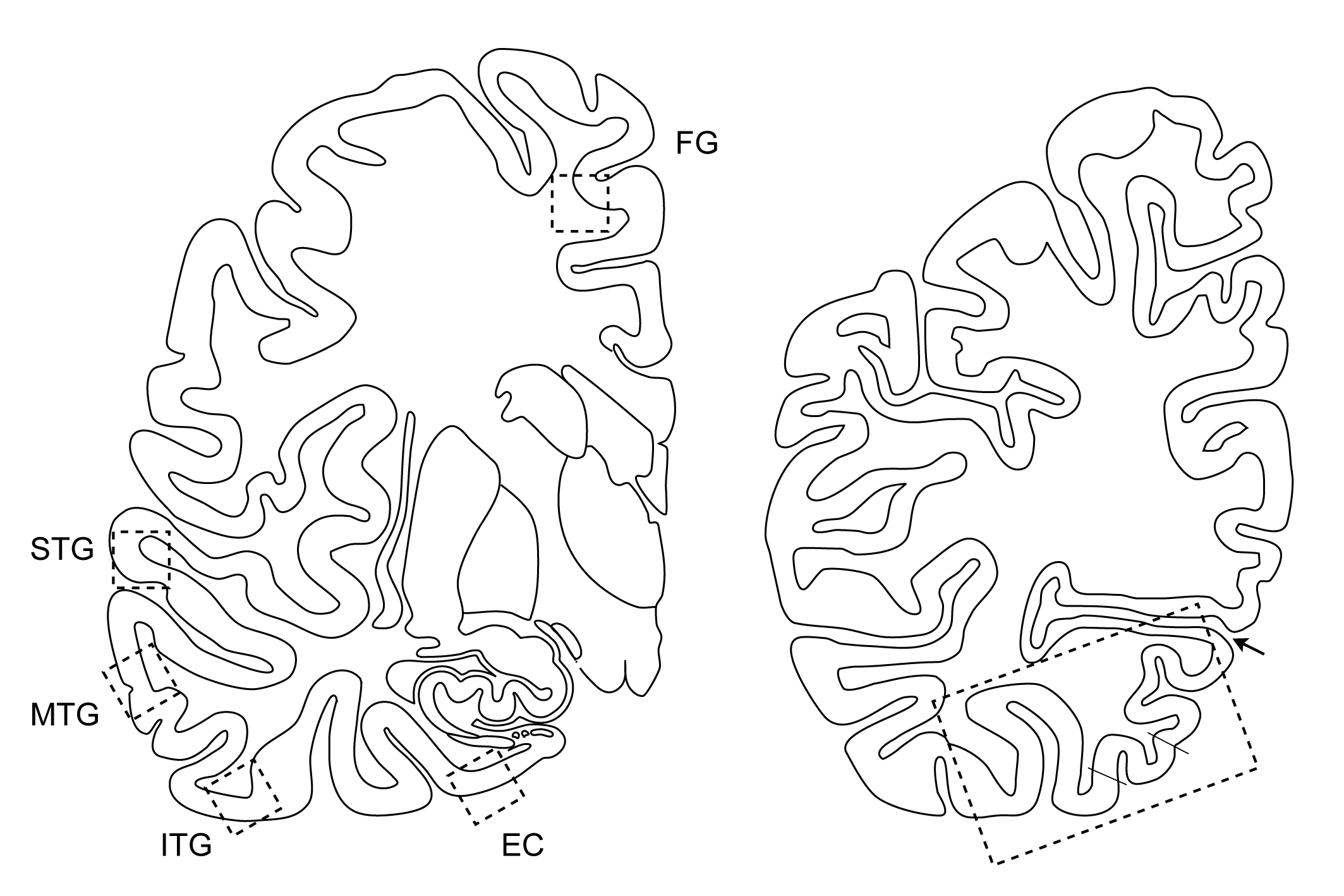

Supplement: Supplementary file 4 — Supplementary Material 4. [file 40478_2024_1792_MOESM4_ESM.tif]

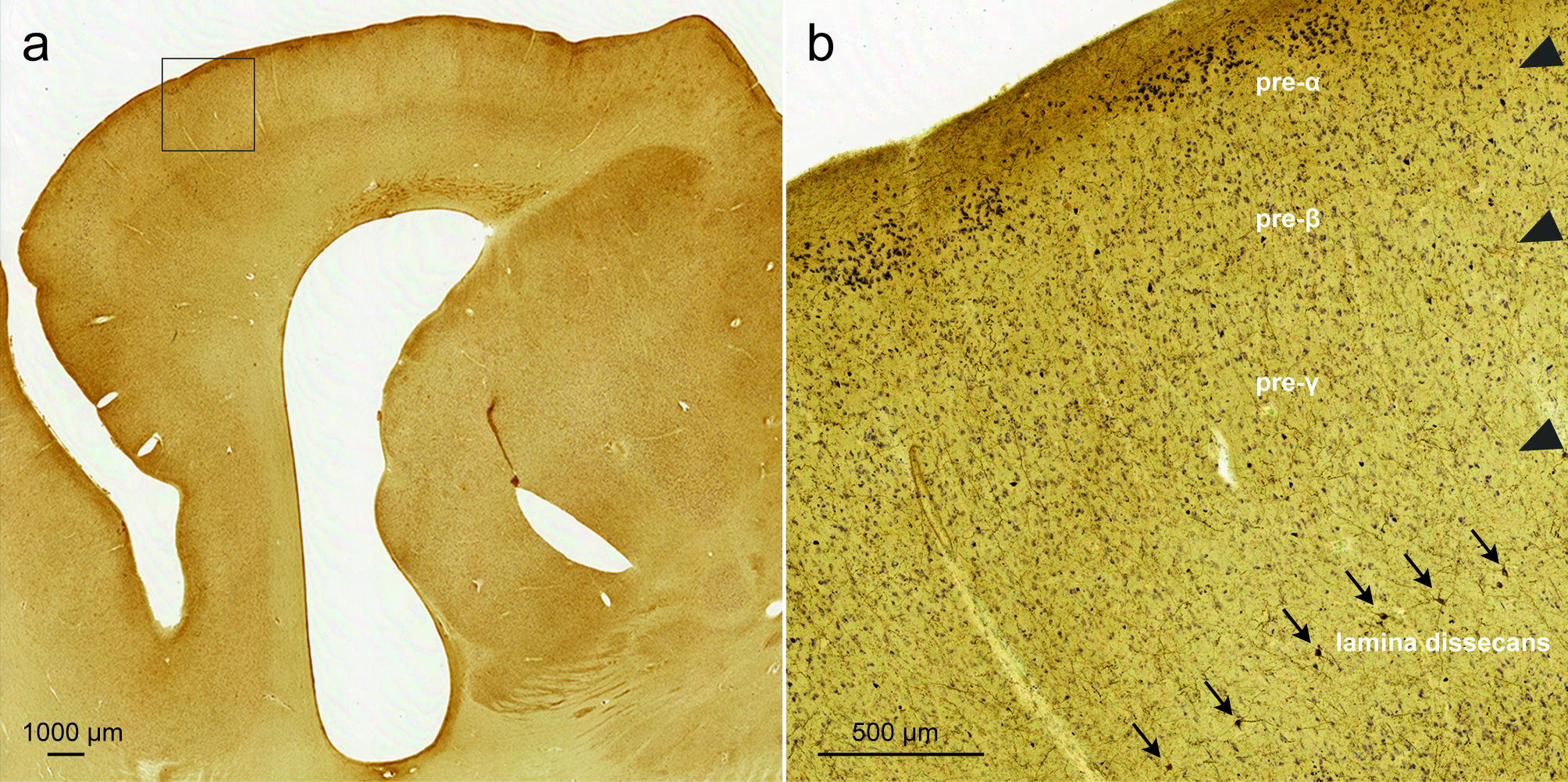

Supplement: Supplementary file 5 — Supplementary Material 5. [file 40478_2024_1792_MOESM5_ESM.tif]

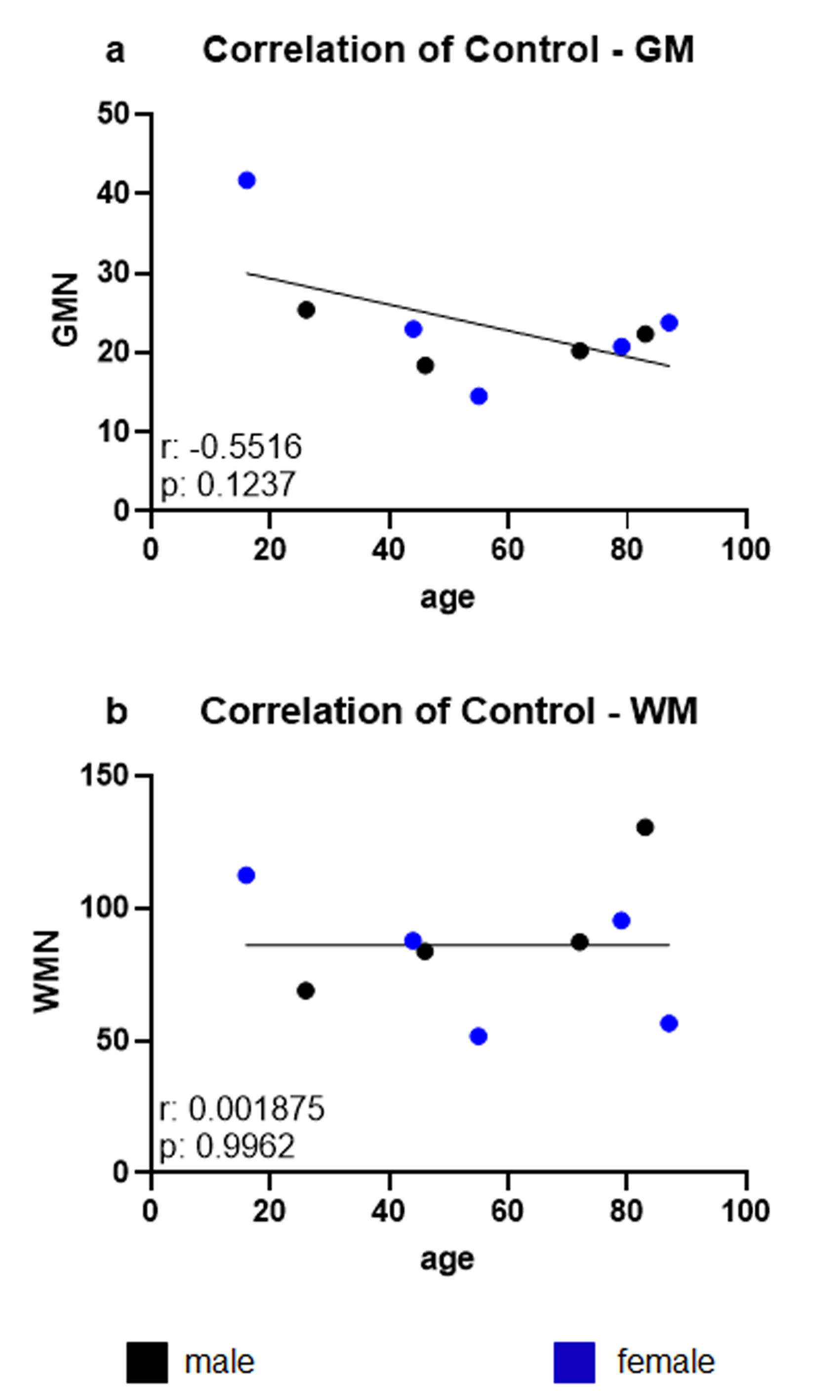

Supplement: Supplementary file 6 — Supplementary Material 6. [file 40478_2024_1792_MOESM6_ESM.tif]

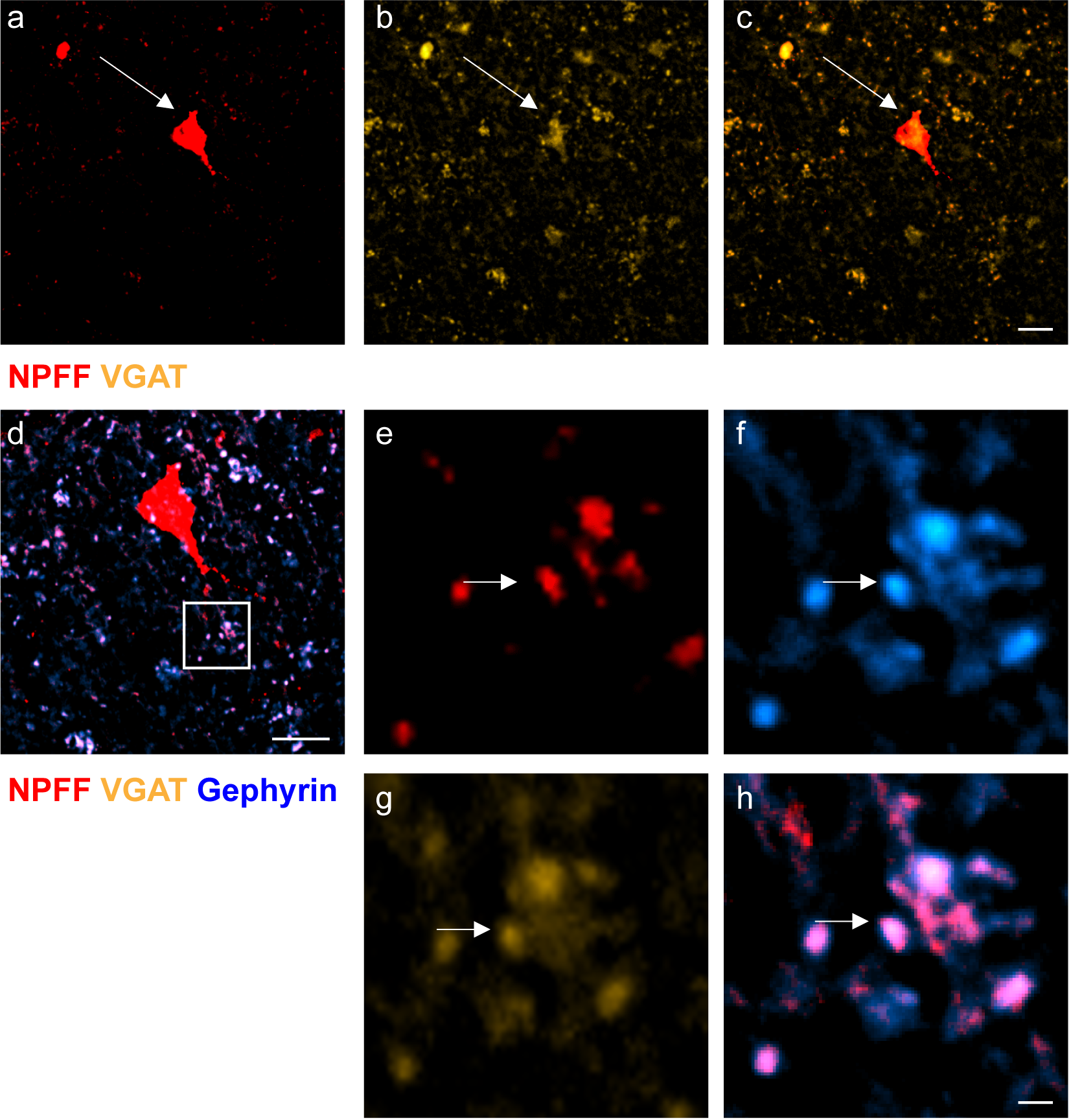

Supplement: Supplementary file 7 — Supplementary Material 7. [file 40478_2024_1792_MOESM7_ESM.tif]
